# The glutamate receptor‐like GLR2.7 modulates insect egg‐induced defense responses in Arabidopsis

**DOI:** 10.1111/nph.70405

**Published:** 2025-07-26

**Authors:** Maria Mineiro, Raphaël Groux, Caroline Gouhier‐Darimont, Pierre Mateo, Christelle Aurélie Maud Robert, Philippe Reymond

**Affiliations:** ^1^ Department of Plant Molecular Biology University of Lausanne Lausanne 1015 Switzerland; ^2^ Institute of Plant Sciences University of Bern Bern 3013 Switzerland

**Keywords:** Arabidopsis, calcium influx, genome‐wide association study, GLR2.7, glutamate, hypersensitive‐like response, insect eggs, *Pieris brassicae*, salicylic acid

## Abstract

- Upon perception of insect eggs, *Arabidopsis thaliana* activates a generic immune response that culminates in cell death (hypersensitive‐like response (HR‐like)). While this response can subsequently impact egg survival, the molecular mechanisms are poorly understood.
- Through a genome‐wide association study (GWAS), we identified the amino acid‐gated calcium channel *GLUTAMATE‐LIKE RECEPTOR2.7* (GLR2.7) as an important gene controlling the extent of HR‐like and accumulation of salicylic acid (SA) in response to egg extract of *Pieris brassicae*. Analysis of natural polymorphisms showed that two major haplotypes segregate at the species‐wide level and suggests that balancing selection acts at this locus.
- Insect oviposition triggered a long‐lasting localized cytosolic calcium accumulation that depended on GLR2.7 and was linked with egg‐associated glutamate (Glu).
- We propose that Glu‐activated GLR2.7 is involved in egg perception and early immune responses.

## Introduction

Plants face various biotic threats in nature and have evolved an efficient immune system that relies on the perception of enemy‐associated molecules and activation of defenses. In particular, the study of plant–insect interactions has been the focus of intense research in the past decades, and a wealth of information on signaling steps and molecular players has accumulated (Erb & Reymond, 2019). By contrast, knowledge on how plants respond to the perception of seemingly inert insect eggs at the molecular level is still limited. Oviposition triggers defense responses in different plant species after recognition of egg‐associated molecules (Hilker & Fatouros, 2015, 2016; Reymond, 2022). They include the production of ovicidal substances, neoplasm formation, egg‐crushing tissue outgrowth, or attraction of egg parasitoids (Reymond, 2013; Hilker & Fatouros, 2015). In plants of the Brassicales order, an efficient defense response is a localized cell death underneath eggs or at the site of egg extract (EE) application, a process named hypersensitive‐like response (HR‐like) based on the resemblance to the effector‐triggered immunity (ETI) response triggered by adapted pathogens (Reymond, 2013; Hilker & Fatouros, 2016). Several studies have reported that HR‐like symptoms induced by eggs from lepidopteran Pieridae species can result in egg desiccation, increased egg mortality, higher egg parasitism, and lower subsequent larval performance (Shapiro & DeVay, 1987; Pashalidou *et al*., 2013; Fatouros *et al*., 2014; Griese *et al*., 2017). Furthermore, the intensity of *Pieris brassicae* egg‐induced HR‐like and the extent of defense gene induction was shown to vary between or within Brassicaceae species (Griese *et al*., 2021; Groux *et al*., 2021; Bassetti *et al*., 2022, 2024; Caarls *et al*., 2023), indicating that this trait is under genetic control. Indeed, three loci containing cell‐surface receptor‐like kinases (RLK), TIR‐NBS‐LRR (TNL) intracellular receptors, and genes involved in innate immunity were associated with HR‐like in *Brassica rapa* (Bassetti *et al*., 2022). A single locus containing a cluster of TNLs was shown to control HR‐like in *Brassica nigra* (Bassetti *et al*., 2024). However, Arabidopsis symptoms in response to oviposition are primarily chlorosis or mild cell death, reminiscent of the pattern‐triggered immunity (PTI), an innate immunity response that follows detection of microbial patterns. By contrast, *B. nigra* displays a much more intense and egg‐killing response that extends beyond the oviposition site and can be qualified as a typical ETI that may have evolved to counteract yet‐to‐be‐discovered egg‐derived effectors (Caarls *et al*., 2023; Bassetti *et al*., 2024).

Signaling of egg‐triggered immunity in different species is still poorly understood, but the accumulation of reactive oxygen species (ROS) together with salicylic acid (SA) has been frequently observed in plants expressing HR‐like (Little *et al*., 2007; Bruessow *et al*., 2010; Bittner *et al*., 2017; Bonnet *et al*., 2017; Geuss *et al*., 2017; Lortzing *et al*., 2019; Caarls *et al*., 2023). In Arabidopsis, it was shown that treatment with *P. brassicae* eggs or EEs activates PTI, including early induction of defense genes, and that SA‐dependent signaling and biosynthesis are required for HR‐like induction (Little *et al*., 2007; Gouhier‐Darimont *et al*., 2013). Phosphatidylcholines (PCs) in eggs were found to activate these immune responses via the LecRK‐I.1 homolog LecRK‐I.8 (Stahl *et al*., 2020), a receptor that was previously identified as a significant component of egg perception (Gouhier‐Darimont *et al*., 2019). Interestingly, the *lecrk‐I.1* mutant was also impaired in egg‐induced SA accumulation and defense gene expression, suggesting a combined role for these RLKs (Groux *et al*., 2021). Also, PCs were detected in egg‐associated secretions from different herbivores, including *P. brassicae*, and induced PTI responses (Lortzing *et al*., 2024). Nonlipidic and nonproteinaceous components in the *P. brassicae* egg glue induce ROS, callose, ethylene, and HR‐like in *B. rapa* and *B. nigra*, but their exact composition and perception are still unknown (Caarls *et al*., 2023). An annexin (ANN)‐like protein in egg‐associated secretion of the sawfly *Diprion pini* is responsible for the emission of parasitoid‐attracting volatiles in pine, but perception and signaling steps are unknown (Hundacker *et al*., 2022). With the accumulating evidence that hallmarks of PTI are conserved between different plants and against different eggs (Lortzing *et al*., 2019; Lortzing *et al*., 2024), the current model is that, like with microbial pathogens, recognition of conserved insect egg‐associated molecular patterns (EAMPs) leads to a PTI that can trigger defense against further herbivory (Valsamakis *et al*., 2020) or pathogens (Hilfiker *et al*., 2014; Alfonso *et al*., 2021). Some plants that experience more regular oviposition by adapted herbivores may in addition develop ETI to respond to specific effectors (Griese *et al*., 2021; Bassetti *et al*., 2024).

Elevation of cytosolic calcium (Ca^2+^) concentration is a prominent feature of PTI (Köster *et al*., 2022). Similarly, the rise in cytosolic Ca^2+^ plays a role in defense against herbivores through activation of different calcium channels, including cyclic nucleotide‐gated ion channels, glutamate receptor‐like proteins (GLRs), two‐pore channels and ANNs (Mousavi *et al*., 2013; Lenglet *et al*., 2017; Vincent *et al*., 2017; Erb & Reymond, 2019; Meena *et al*., 2019; Malabarba *et al*., 2021). For instance, Arabidopsis GLR3.3 and GLR3.6 are glutamate (Glu)‐gated Ca^2+^ channels that participate in long‐distance wound‐induced plant defenses (Mousavi *et al*., 2013; Toyota *et al*., 2018; Alfieri *et al*., 2019). By contrast, Ca^2+^ signaling in response to oviposition remains poorly characterized. Indirect evidence comes from the observation that eggs from the spider mite *Tetranychus urticae* or from *P. brassicae* induce the expression of Ca^2+^‐related genes in Arabidopsis (Stahl *et al*., 2020; Ojeda‐Martinez *et al*., 2021). However, whether Ca^2+^ accumulation is linked to egg‐induced HR‐like and the nature of calcium channels potentially involved are still unknown.

Exploiting the power of genome‐wide association studies (GWAS) to identify loci underlying genetic variation in Arabidopsis (Atwell *et al*., 2010), we recently explored the response of Arabidopsis accessions to *P. brassicae* EE treatment, which tends to amplify and accelerate the natural oviposition symptoms. Using a robust symptom score ranging from no response to localized chlorosis and cell death, we revealed two loci explaining most of the variation observed, one of them containing *LecRK‐I.1* (Groux *et al*., 2021). We validated the locus by showing that a *lecrk‐I.1* knockout mutant displays decreased HR‐like response after EE treatment and found that two main haplotypes explain part of the variation in this response between Arabidopsis accessions. We also identified potential signatures of balancing selection at this gene, suggesting that it may be important ecologically (Groux *et al*., 2021). Interestingly, a LecRK‐I.1 ortholog was found in a locus associated with HR‐like in *B. rapa* (Bassetti *et al*., 2022).

Here, we investigated the nature of the second locus identified in the GWAS and report that *GLR2.7*, which is part of a small clade 2 GLR cluster located on this locus, plays a significant role in triggering Ca^2+^ accumulation and HR‐like underneath the eggs.

## Materials and Methods

### Plant materials and growth conditions

*Arabidopsis thaliana* (L.) Heynh. plants were grown in growth chambers in short day conditions (10 h light, 100 μmol m^−2^ s^−1^, 22°C, 65% relative humidity) and were 4 to 5 wk old at the time of treatment. For Ca^2+^ measurements, YELLOW CHAMELEON 3.6 (YC3.6) plants were grown *in vitro*. Seeds were surface‐sterilized and then plated on ½‐strength Murashige & Skoog medium (½MS), 0.3% sucrose, and 0.8% agar (w/v). Plates were placed in short day conditions for 10 d, and seedlings were transferred to water in 96‐well plates the day before the experiment. *Nicotiana benthamiana* Domin. seeds were sown directly on soil and cultivated under long day conditions (16 h light, 25°C, 65% relative humidity). A population of the Large White butterfly, *Pieris brassicae* L., was maintained on *Brassica oleracea var. gemmifera* DC (Reymond *et al*., 2000).

### Arabidopsis mutant lines

*GLR2.7* (At2g29120) was mutated in Arabidopsis by a CRISPR‐Cas9 approach. Two single‐guide RNAs, sg1 (cc_GLR_7b_rev) and sg2 (cc_GLR_7a_fw), were inserted into the pDE42‐Cas9, generating pDEGLR2.7. This construct was used to induce a 424‐bp deletion in the *GLR2.7* gene (Supporting Information Fig. S1a). After plant transformation, the presence of a deletion was verified by polymerase chain reaction (PCR), using primers CCglr2.7fw2 and CCglr2.7rv2 flanking the deletion and amplifying a 242‐bp fragment. To generate the beta‐glucuronidase (GUS) reporter line GLR2.7p:NLS‐GFP‐GUS, a 2107‐bp promoter fragment of *GLR2.7* was amplified using the primers GLR2.7pfw and GLR2.7prv. The amplified sequence was cloned upstream of the NLS‐GUS coding sequence in the pMK7S*NFm14GW (VIB, Ghent) vector via the Gateway cloning system. Final expression clones were inserted in *Agrobacterium tumefaciens* strain GV3101 for transformation of Columbia (Col‐0) Arabidopsis by floral dipping.

**Fig. 1:**
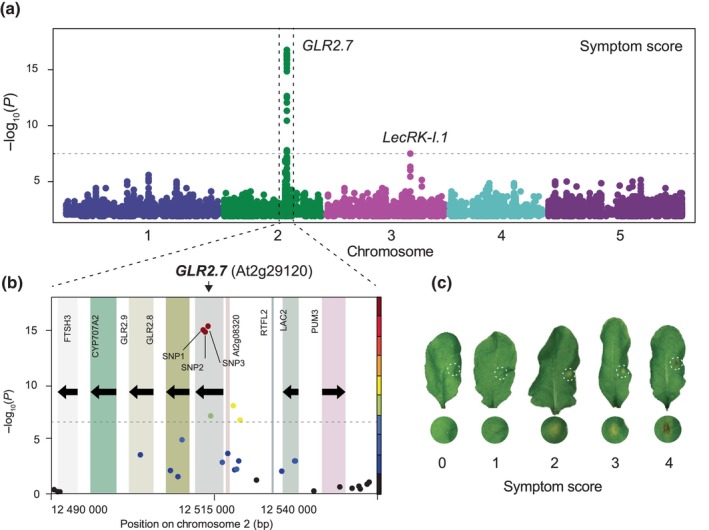
Genome‐wide mapping of insect egg‐induced cell death. (a) Manhattan plot of genome‐wide association study (GWAS) mapping for symptom score after 5 d of *Pieris brassicae* egg extract (EE) treatment of Arabidopsis plants using an accelerated mixed‐model. Full imputed genotypes for all 295 accessions were used for mapping. Chromosomes are displayed in different colors, and the horizontal dashed line indicates the Bonferroni‐corrected significance threshold at *α* = 0.05. (b) Local association plot of the *GLUTAMATE‐LIKE RECEPTOR2.7* (*GLR2.7*) locus using the 250 K genotype data for symptom score. The *x*‐axis represents genomic position on Chromosome 2, and color boxes indicate genes. Linkage disequilibrium (LD) with the most significant single nucleotide polymorphism (SNP) is indicated by a color scale. The dashed line indicates the Bonferroni corrected significance threshold at *α* = 0.05. (c) Representative images of EE‐treated (dashed circles) leaves with symptoms used for scoring accessions. Shown below are zoomed images of the site of EE treatment.

Arabidopsis accessions were categorized as ‘weak’ or ‘strong’ depending on whether their symptom score in response to EE was in the lower, respectively higher, quintile (Groux *et al*., 2021; Fig. 1; Table S1). Complementation of ‘weak’ accessions was performed using the *GLR2.7* genomic sequences from ‘strong’ accessions. *GLR2.7* sequences from Tomegap‐2 (N76250) and Lz‐0 (N28482) were amplified and cloned into the plasmid pGreen229mVenus, using the primers GLR2.7‐clon‐fw and GLR2.7‐clon‐rv, and digested with EcoRV and BamHI. pGreen229mVenus_GLR2.7^Tomegap‐2^ and pGreen229mVenus_GLR2.7^Lz‐0^ were agroinfiltrated into ‘weak’ accessions, Ull2‐3 (N78817) and Ren‐1 (N77210), respectively. In addition, Ull2‐3 was complemented with the *GLR2.7* genomic sequences from Ren‐1 (pK7_Ren1GLR2.7) and Ull2‐3 (pK7_Ull23GLR2.7), using the Gateway cloning with primers GLR2.7‐clon‐Fw2 and GLR2.7‐5UTR‐Rv for the promoters, and primers GLR2.7‐ATG‐Fw and GLR2.7‐clon‐Rv for the GLR2.7 gene sequences.

Arabidopsis lines (MCGf and MCGf_*glr2.7*) used for calcium quantification were obtained by transformation of Col‐0 and *glr2.7* with the plasmid pB7_UBQ10_CGf. pB7_UBQ10_CGf was obtained after amplification of the region of interest of TCTPpro_UBQ10p_mCherry_GCaMP6f (Weigand *et al*., 2021) using the primers attB1_Fragment_FOR and GCaMP6‐rv and cloned into pB7m24GW3 (VIB, Ghent, Belgium) under the ubiquitin‐10 (UBQ10) promoter using the Gateway system. The resulting expression construct (pB7_UBQ10_CGf) was inserted in *A. tumefaciens* GV3101 for transformation of Arabidopsis by floral dipping.

The SA‐biosynthesis mutant *sid2‐1* was obtained from C. Nawrath (University of Lausanne). T‐DNA insertion lines for *glr2.7* (SALK_121990), *glr2.8* (SALK_111659) and *glr2.9* (SALK_125496) were obtained from the NASC stock center. The CRISPR‐Cas9‐generated triple mutant *glr2.7/2.8/2.9* was obtained from C. Zipfel (University of Zürich) and described previously (Bjornson *et al*., 2021).

### Calcium and glutamate reporter lines

Ca^2+^ fluxes were monitored using Arabidopsis GCaMP6f and YC3.6 reporter lines. For experiments measuring calcium in *glr2.7*, we used the lines MCGf and MCGf_*glr2.7*. The ratiometric CGf line (mCherry fused to GCaMP6f) was provided by A. Costa (Weigand *et al*., 2021). The YC3.6 in Col‐0 and *glr2.7/2.8/2.9* background was obtained from C. Zipfel (University of Zurich). The 35S::CHIB‐iGluSnFR Glu reporter line (Toyota *et al*., 2018) was obtained from C. Faulkner (John Innes Center, Norwich, UK).

### Oviposition and treatment with EE

Plants were placed in a tent containing *c*. 20 *P. brassicae* butterflies for a maximum of 2 h and then placed in a growth chamber in plastic boxes until hatching of the eggs. Control plants were kept in the same conditions without butterflies. *P. brassicae* eggs were collected and crushed with a pestle in Eppendorf tubes. After centrifugation (15 000 ***g***, 3 min), the supernatant (‘egg extract’) was collected and stored at −20°C. For each plant, two leaves were treated with 2 μl of EE. A total of four plants were used for each experiment. After 3 to 6 d, depending on the experiment, EE was gently removed with a scalpel blade and treated leaves were stored in liquid nitrogen. Untreated plants were used as controls. For calcium measurement using YC3.6, plants were grown *in vitro* and used at 11 d. EE was added to 96‐well plates containing seedlings at a final dilution of 1/150.

### Glutamate treatment

L‐glutamic acid (G1251; Sigma‐Aldrich), ≥ 99% (high‐performance liquid chromatography (HPLC)) was prepared at a 58 mM stock solution in water. For experiments with 4‐ to 5‐wk‐sold plants, a 2 μl drop of 10 mM Glu, diluted in a control solution (0.05% Silwet L‐77), was applied to the abaxial leaf surface.

### Genome‐wide association mapping and haplotype analysis

GWAS analysis of Arabidopsis response to insect eggs was described recently (Groux *et al*., 2021). Briefly, a set of 295 accessions from the HapMap panel (Horton *et al*., 2014) was used. For each accession, three leaves from three to six plants were treated with EE diluted 1 : 1 with deionized water. Treated plants were left in the growth chamber for an additional 5 d until phenotyping. After 5 d, treated leaves were removed with forceps, symptoms were scored from 0 to 4, which are arbitrary units corresponding to the intensity of the response to EE (Groux *et al*., 2021; Fig. 1; Table S1), and leaves were frozen in liquid nitrogen for further SA quantification. Pools of 30 accessions were phenotyped every week. GWAS mapping, haplotype analysis, and population‐wide cladogram were described previously (Groux *et al*., 2021). Haplotype networks were built using the ‘ape’ and ‘pegas’ R packages on the full GLR2.7 gene from 122 sequenced accessions. Sequences were obtained from SALK 1001 genome browser (http://signal.salk.edu/atg1001/3.0/gebrowser.php), and sequences were aligned using Multiple Alignment using Fast Fourier Transform for further analysis.

### Cell death measurement

For visualization of cell death, EE was gently removed. For all experiments, cell death was quantified by red light measurements in Arabidopsis leaf disks at 6 d posttreatment using a Hidex microplate reader (excitation at 650 nm and emission at 680 nm; Landeo Villanueva *et al*., 2021).

### Salicylic acid quantifications

SA quantification was performed using the bacterial biosensor *Acinetobacter* sp. ADPWH using a Hidex microplate reader, according to the published protocols (Huang *et al*., 2006; Stahl *et al*., 2020; Groux *et al*., 2021).

### Gene expression analysis

Analysis of gene expression by real‐time quantitative PCR was described previously (Groux *et al*., 2021). For analysis of *GLR2.7* expression in different Arabidopsis accessions, primers were designed to match *GLR2.7* sequences from all accessions. A list of all primers used in this study is found in Table S2.

### Transient transformation of *N. benthamiana* and protein visualization

To visualize and determine the subcellular localization of the GLR2.7 protein *in planta*, the construct 5′ untranslated region (UTR)‐coding sequence of GLR2.7^Col‐0^‐Venus‐3'UTR was synthesized by GenScript, amplified using the primers GLR2.7‐5UTR‐Fw and GLR2.7‐clon‐Rv, and cloned into pB7m34GW (VIB) under the ubiquitin‐10 (UBQ10) promoter using the Gateway system. The resulting expression construct (pB7UBQ10_GLR2.7_Venus) was inserted in *A. tumefaciens* GV3101 for leaf infiltration in 4‐wk‐old *N. benthamiana* plants. Epidermal cells of infiltrated leaves were observed 2 d postinfiltration. To visualize the plasma membrane, an aqueous solution of 10 μM FM4‐64 (SymaptoRed Reagent; Sigma‐Aldrich, 574 799, ≥ 99%, HPLC) was infiltrated into the target leaf area 15 min before imaging.

### Fluorescence microscopy

For analysis of GCaMP6f and iGluSnFR reporter lines, imaging of leaf surfaces was conducted using an SMZ18 stereomicroscope equipped with an ORCA‐Flash4.0 camera and eGFP and mCherry emission/excitation filter sets.

For GLR2.7‐mVenus protein localization, fluorescence imaging of epidermal cells was performed using a Leica Stellaris confocal laser scanning microscope. Images were acquired with a 20×/0.75 dry objective at zoom factor 5.

Excitation and detection windows were set as follows: eGFP (470 nm, 510–523 nm), mVenus (514 nm, 520–550 nm), and mCherry (561 nm, 605–650 nm). All images were processed using the imagej software (v.2.14.0/1.54f).

### Amino acid quantification

To quantify amino acids released from *P. brassicae* eggs, butterflies were allowed to oviposit on filter paper as described previously (Stahl *et al*., 2020). Eggs and filter papers were collected separately after 1 d. Samples were immediately flash‐frozen in liquid nitrogen and stored at −80°C until analysis. Extraction buffer (EtOH/H_2_O, 50 : 50, 0.1% HCO_2_H) was added to each sample at 1 : 10, w/v. Samples were centrifuged for 10 min at 10 400 ***g*** at 10°C, and supernatants were separated and evaporated at 45°C under vacuum. The residues were reconstituted with ultra‐pure water, resulting in a 10‐fold concentration (e.g. 200 μl supernatant reconstituted in 20 μl water). Free amino acids were quantified using the AccQ‐Tag Ultra Derivatization Kit (Waters, Milford, MA, USA) according to the manufacturer's instructions. Amino acid content was analyzed by liquid chromatography mass spectrometry (LC‐MS) in positive mode with single‐ion recording and quantified by comparison with a mixture of the corresponding standards (Meier *et al*., 2024).

### Cytosolic calcium and apoplastic glutamate analyses

GCaMP6f and iGluSnFR reporter lines were treated with EE or Glu on the abaxial side of the leaf. Images were acquired at 0, 10, 30, and 60 min as well as 24, 48, and 72 h posttreatment. Fluorescence intensity in the treated area was quantified using the imagej2 software (v.2.14.0/1.54f), and the ratio *R* = GFP/mCherry was calculated. The relative change of fluorescence was then determined as (*R*–*R*_0_)/*R*_0_, where *R*_0_ is the ratio value at time 0. For background correction, Arabidopsis Col‐0 wild‐type (WT) leaves were treated with EE and imaged at the same time points as the reporter lines. The low autofluorescence signal detected in GFP and mCherry channels in EE‐treated WT leaves was quantified and subtracted from the fluorescence measurements of reporter lines.

For YC3.6 seedlings, plants were placed in black 96‐well plates containing 150 μl of distilled water and kept in the dark for 12 h. The following day, the water was carefully replaced without disturbing the seedlings; the signal acquisition and the data analysis were performed as described in Bjornson *et al*. (2021).

### Data analysis

Statistical analyses were performed using graphpad prism 10 (v.10.1.1). GWAS mapping and subsequent analysis of the data obtained were performed with the R software v.3.6.

## Results

### GWAS mapping and identification of associated loci

To investigate the genetic basis of insect egg‐induced HR‐like responses, we performed a GWAS on a world‐wide set of 295 Arabidopsis accessions. Symptom score (from 0 to 4; Fig. 1) and total SA levels were quantified in all accessions after 5 d of EE treatment and were used for mapping (Table S1). Initial mapping using an imputed genotype matrix for 2029 accessions (Togninalli *et al*., 2018) revealed a highly significant peak associated with symptom score and total SA levels (−log_10_(*P*) = 15.96 and 10.64, respectively) spanning over 10 Kb on Chromosome 2 (Figs 1, S2 for SA) and one marker reaching significance on Chromosome 3 (−log_10_(*P*) = 7.59) that is only associated with symptom score. The description of this second locus was previously reported and revealed that the receptor kinase LecRK‐I.1 (At3g45330) contributes significantly to insect egg‐induced responses (Groux *et al*., 2021). As the genotype matrix used was produced by imputing missing genotypes based on a subset of accessions that were sequenced, we used the 250 K single nucleotide polymorphism (SNP) genotype data that were available for all used accessions for further investigation of specific markers (Horton *et al*., 2012). The three most significantly associated SNPs occurred within the coding region of GLR2.7 for both phenotypes, while two additional markers were located in the promoter of the gene (Figs 1, S2). Interestingly, this locus contains two other clade 2 GLR members, *GLR2.8* and *GLR2.9*, which are the closest homologs of *GLR2.7* (Roy & Mukherjee, 2017). To further explore whether variation within *GLR2.7* could be causal for variation in HR‐like responses and SA accumulation, we examined linkage disequilibrium (LD) between the top SNP and the other markers in a 50‐Kb window around the variant. We found that the most significantly associated SNPs in both scans were in very high LD with other SNPs located in the gene body of GLR2.7, while LD decayed rapidly around the gene for other markers (Figs 1b, S2). Both SNP1 and SNP2 appeared to be in very high LD with SNP3, suggesting that this gene might be involved in cell death induction and SA accumulation upon insect egg perception.

As mentioned, SA is necessary to induce cell death upon EE perception (Gouhier‐Darimont *et al*., 2013). Interestingly, we found that total induced SA and symptom score were moderately correlated (*r* = 0.40) in the entire mapping panel (Fig. S2). This indicates that SA does not fully explain the strength of egg‐induced HR‐like responses and that other signaling components might also contribute, which is consistent with the partial reduction in cell death observed in the SA‐biosynthesis mutant *sid2‐1* (Gouhier‐Darimont *et al*., 2013). However, this could also be consistent with SA working in a more qualitative way, as almost all accessions showed some degree of SA accumulation in response to EE treatment, regardless of the degree of symptoms (Table S1).

### Validation of the GLR2.7 locus

**Fig. 2:**
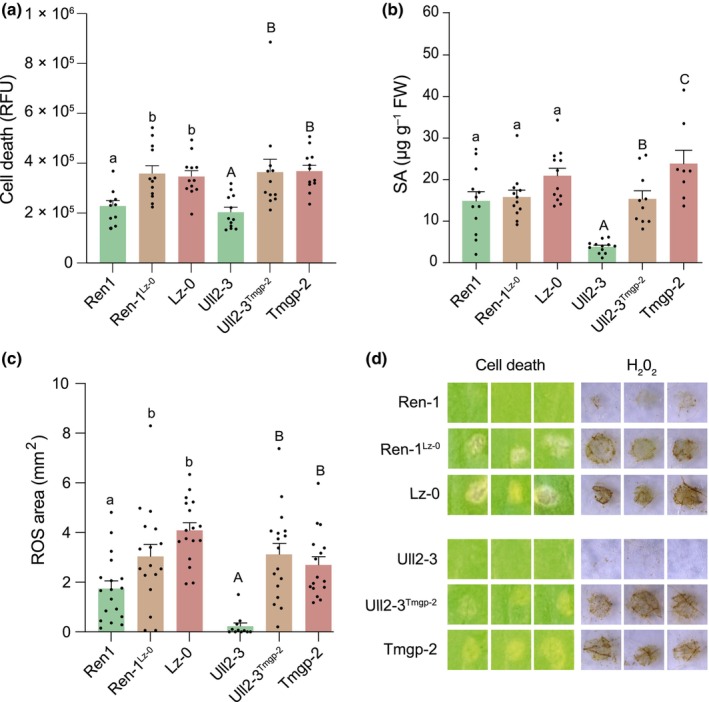
Arabidopsis *GLUTAMATE‐LIKE RECEPTOR2.7 (GLR2.7)* locus is responsible for quantitative variation in *Pieris brassicae* egg extract (EE)‐induced responses. Complementation of Ren‐1 and Ull2‐3 ‘weak’ *Arabidopsis thaliana* accessions with *GLR2.7* from Lz‐0 and Tomegap‐2 (Tmgp‐2) ‘strong’ accessions leads to enhanced cell death (a, d), salicylic acid (SA) accumulation (b), and H_2_0_2_ accumulation (c, d) in response to EE treatment. (a) Cell death quantification after 6 d of EE treatment was measured by red light fluorescence. Mean ± SE of one biological replicate is shown (*n* = 10–12), this experiment was repeated twice with similar results. (b) SA quantification after 3 d of EE treatment. Mean ± SE of three biological replicates is shown (*n* = 4 per experiment). (c) H_2_0_2_ accumulation measured by 3,3′‐Diaminobenzidine (DAB) staining after 3 d of EE treatment. Mean ± SE of one biological replicate is shown; this experiment was repeated twice with similar results. (d) Representative pictures of cell death and H_2_O_2_ accumulation (DAB staining), respectively after 6 and 3 d of EE treatment. Statistical differences within each group of weak, complemented, and strong accessions are indicated by lowercase and upper‐case letters (ANOVA followed by Tukey's HSD). For clarity, weak accessions are depicted by green bars, strong accessions by red bars and complemented accessions by brown bars. RFU, relative fluorescence unit; ROS, reactive oxygen species.

To verify that the *GLR2.7* locus identified in the GWAS was responsible for the observed phenotype, we first selected accessions that showed a highly contrasting response to EE treatment. Ren‐1 and Ull2‐3 were among the 10 accessions with the lowest score (< 0.06) and were considered ‘weak’ accessions, whereas Lz‐0 and Tomegap‐2 were among the top 20 accessions with the highest symptom score (> 3.00) and were considered ‘strong’ accessions (see the Materials and Methods section and Table S1). Then, we introduced the *GLR2.7* genomic sequence from strong accessions into weak accessions. Strikingly, when *GLR2.7* from Lz‐0 and Tomegap‐2 was introduced into Ren‐1 and Ull2‐3, respectively, each complemented accession showed an HR‐like that was significantly higher than in the weak accession and as high as in the strong accession (Fig. 2a,d). Also, the EE‐induced SA level in Ull2‐3 complemented with *GLR2.7* ^*Tomegap‐2*^ was significantly higher than in Ull2‐3 (Fig. 2b). However, *GLR2.7* ^*Tomegap‐2*^ did not fully restore SA accumulation in Ull2‐3 and this effect was not observed for *GLR2.7* ^*Lz‐0*^ in Ren‐1, suggesting a partial contribution of GLR2.7 for this response.

Given the known correlation between egg‐induced HR‐like and ROS accumulation (Little *et al*., 2007; Bittner *et al*., 2017; Geuss *et al*., 2017), we then measured EE‐induced H_2_O_2_ in the complemented accessions. Again, introducing GLR2.7 from strong into weak accessions significantly enhanced ROS production (Fig. 2c,d).

In addition, we complemented the weak Ull2‐3 with *GLR2.7* from the weak accessions Ull2‐3 or Ren‐1, and from the strong accession Tomegap‐2 as a positive control. Only *GLR2.7* ^*Tomegap‐2*^ was able to increase HR‐like when introduced in Ull2‐3, indicating that this effect is specifically due to the *GLR2.7* haplotype (Fig. S3). Collectively, these data confirm that the *GLR2.7* locus is responsible for the observed variation in EE‐induced HR‐like and SA content between Arabidopsis accessions.

**Fig. 3:**
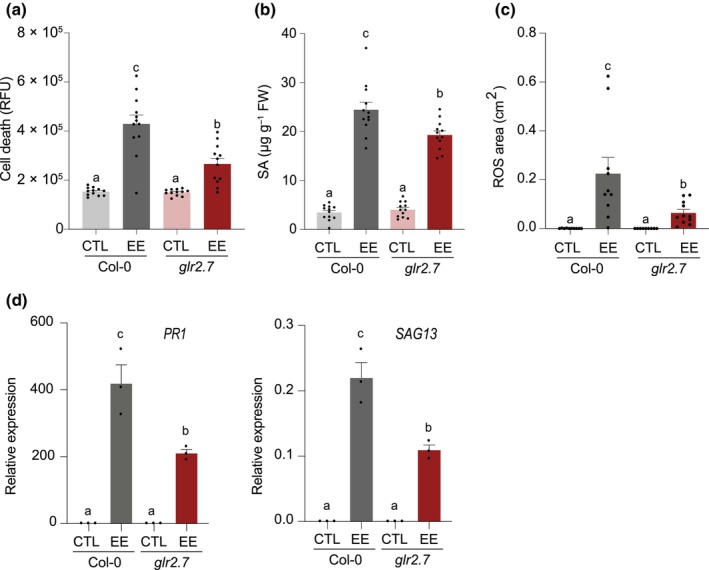
Arabidopsis *GLUTAMATE‐LIKE RECEPTOR2.7 (*GLR2.7) modulates responses to egg extract (EE). (a) Cell death quantification after 6 d of *Pieris brassicae* EE treatment was measured by red light fluorescence. Mean ± SE of one biological replicate is shown (*n* = 12). This experiment was repeated twice with similar results. (b) Salicylic acid (SA) quantification after 3 d of EE treatment. Mean ± SE of three biological replicates is shown (*n* = 4 per experiment). (c) H_2_0_2_ accumulation measured by DAB staining after 3 d of EE treatment. Mean ± SE of one biological replicate is shown (*n* = 10–13). This experiment was repeated twice with similar results. (d) Expression of egg‐induced defense marker genes after 3 d of EE treatment. Transcript levels were monitored by real‐time quantitative polymerase chain reaction and normalized to the reference gene SAND. Mean ± SE of three technical replicates is shown. This experiment was repeated twice with similar results. Letters denote statistical differences (ANOVA followed by Tukey's HSD). ROS, reactive oxygen species.

To further investigate the role of the *GLR2.7* locus in the quantitative response to EE, we generated a *GLR2.7* ^Col‐0^ knockout by CRISPR‐Cas9. In this *glr2.7* mutant, no measurable expression could be detected (Fig. S1b). Importantly, *glr2.7* displayed significantly lower EE‐induced HR‐like, SA accumulation, and ROS production compared with Col‐0 (Fig. 3a–c), indicating that the modulation of these responses depends on a functional protein. Expression of the known egg‐responsive genes *PR1* and *SAG13* (Stahl *et al*., 2020) was also attenuated in *glr2.7* (Fig. 3d).

In summary, we identify GLR2.7 as a modulator of egg‐induced defense responses. This protein contributes to the quantitative variation in HR‐like and SA levels between accessions, together with LecRK‐I.1 for HR‐like (Groux *et al*., 2021).

### Different haplotypes of GLR2.7 segregate in Arabidopsis populations

**Fig. 4:**
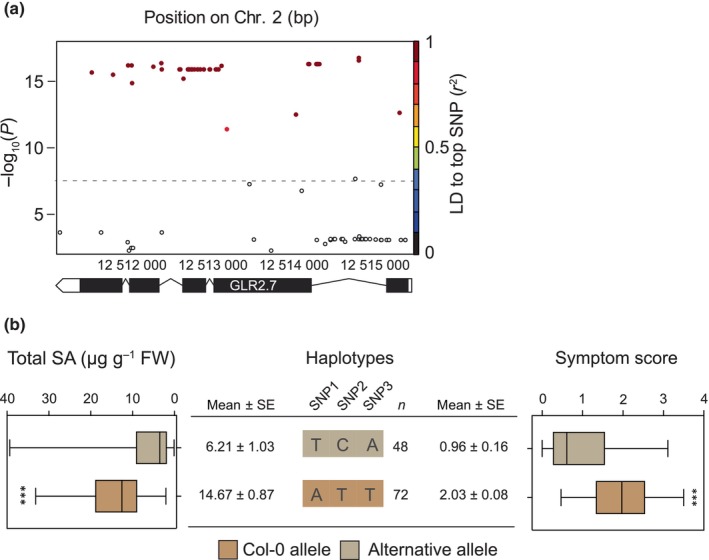
Local association and haplotype analysis of the Arabidopsis *GLUTAMATE‐LIKE RECEPTOR2.7 (GLR2.7)* locus. (a) Local association plot of the *GLR2.7* locus using the full imputed genotype data. The *x*‐axis represents genomic position on Chromosome 2. Linkage disequilibrium (LD) with the most significant SNP is indicated by a color scale. The dashed line indicates the Bonferroni‐corrected significance threshold at *α* = 0.05. (b) The three selected SNPs from Fig. 1 define two major haplotypes (only haplotypes containing at least two accessions are shown). Min and Max values are indicated, as well as the median (horizontal line). The box represents 50% of the values. The whiskers extend from the box to the minimal and maximal values. Box and whisker plots of total salicylic acid (SA) and symptom score after 5 d of egg extract (EE) treatment are shown. Significant differences are indicated (Student's *t*‐test, ***, *P* < 0.01). Col‐0, Columbia.

A deeper analysis of the *GLR2.7* locus using the imputed genotype matrix for 2029 accessions revealed different haplotypes segregating in the population. By looking at all significantly associated markers within the *GLR2.7* gene sequence, we found 49 SNPs spanning the entire coding sequence and promoter, and most of them showed a highly significant association with symptom score (−log_10_(*P*) > 10; Fig. 4a). As with the lower density SNP data, we found that all markers within the *GLR2.7* coding region were in very high LD (> 0.8). The fact that many markers are genetically linked again supports the potential existence of distinct haplotypes segregating at this locus. Overall, out of 49 significantly associated SNPs, 16 were found to induce nonsynonymous changes in the protein sequence. Regarding SNP1‐3, SNP1 is located in an intron, SNP2 leads to a synonymous amino acid change, and SNP3 to the nonsynonymous substitution V448D (Tables S1, S3).

**Fig. 5:**
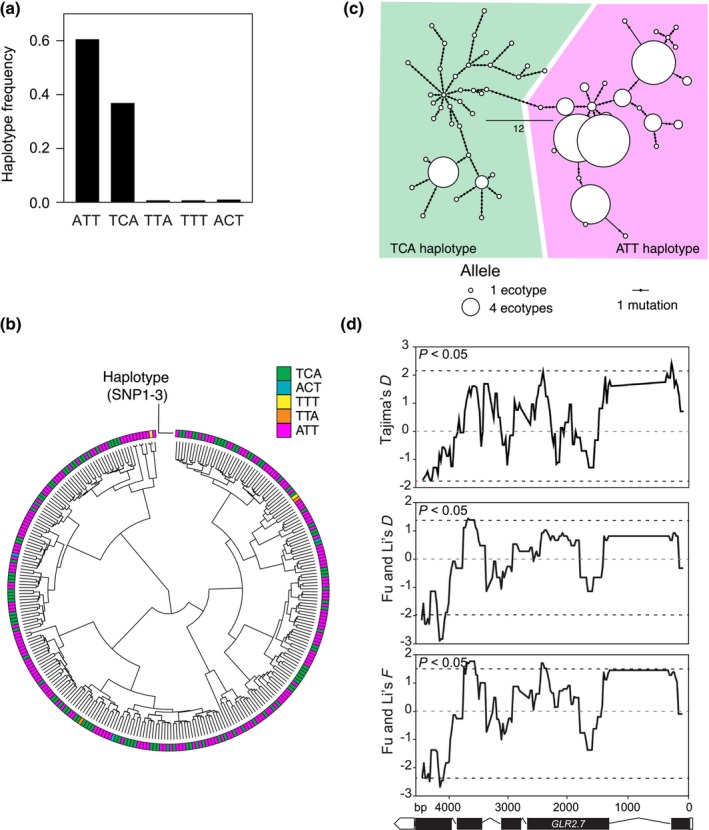
Various signatures of balancing selection at the Arabidopsis *GLUTAMATE‐LIKE RECEPTOR2.7 (GLR2.7)* locus. (a) Frequency of the haplotypes within a world‐wide set of Arabidopsis accessions. (b) Cladogram constructed from a genome‐wide kinship matrix of the 295 accessions used in this study. The outermost circle indicates haplotype. (c) Haplotype network of the *GLR2.7* sequence in the 122 sequenced accessions. Circles represent haplotypes, and lines represent the number of stepwise mutations separating two haplotypes. The two colored areas delimit sequences belonging to the haplotypes defined using genome‐wide association study (GWAS) data. (d) Sliding window analysis of Tajima's *D*, Wu and Li's *D* and *F* statistics along the *GLR2.7* coding region using a window size of 200 bp and a step size of 25 bp. A subset of 125 accessions for which available full genome sequences were used for this analysis. For Wu and Li's statistical tests, *Arabidopsis lyrata* was used as an outgroup. The dashed lines indicate significance threshold at *P* < 0.05. The gene structure of *GLR2.7* is shown below.

To evaluate the population‐wide genetic structure of this locus, we used the three significant SNPs (SNP1‐SNP3) identified in Fig. 1 to search for *GLR2.7* haplotypes. In most accessions, SNP1 was either a T or an A, SNP2 either a C or a T, and SNP3 either an A or a T (Table S1). Strikingly, we found that SNP1‐SNP3 define two main haplotypes within the entire mapping population, one having TCA alleles for SNP1‐SNP2‐SNP3 and associated with a low symptom score (weak HR‐like) and another one having ATT alleles and associated with a high symptom score (strong HR‐like; Fig. 4b). We thereafter named these two haplotypes *GLR2.7* ^ATT^ and *GLR2.7* ^TCA^, respectively. By contrast, other allelic series were either not present or contained a very low number of accessions and were therefore not further considered (Fig. 5a). Interestingly, Col‐0 possesses the *GLR2.7* ^ATT^ haplotype (Fig. 4b), as well as Lz‐0 and Tomegap‐2 mentioned above. Altogether, our data indicate the existence of alleles at the *GLR2.7* locus that modulates the intensity of cell death and contributes partially to SA accumulation upon treatment with *P. brassicae* EE. We also noticed that, overall, the 49 significant SNPs found in the GLR2.7 locus tend to have only two variants that correspond to either the TCA or the ATT haplotype (Table S1).

### Signatures of balancing selection at the GLR2.7 locus

Genes that confer herbivore or pathogen resistance are advantageous in the presence of pests but are usually costly or detrimental to maintain in their absence (Vila‐Aiub *et al*., 2011; Van Velzen & Etienne, 2015). Balancing selection is a process through which multiple alleles are kept at intermediate frequency in a population, a process that is known to shape disease resistance loci in plants (Bakker *et al*., 2006; Huard‐Chauveau *et al*., 2013). Loci under balancing selection display highly diverged alleles, and haplotype distribution is widespread across the geographical range of the considered species. As shown by the haplotype frequency distribution at the *GLR2.7* locus, two haplotypes defined by SNP1‐3 are present at intermediate frequencies, while all others are found in very few individuals (Fig. 5a).

To explore whether the distribution of *GLR2.7* haplotypes was caused by geographical or phylogenetic factors, we constructed a cladogram representing genome‐wide distance between accessions by using the kinship matrix used for the GWAS mapping. Clearly, both GLR2.7^ATT^ and GLR2.7^TCA^ haplotypes are homogeneously distributed among all populations of Arabidopsis, indicating that they are not confined by phylogenetic or geographical proximity (Figs 5b, S4). To further analyze the genetic structure of *GLR2.7*, we used gene sequences from 122 accessions with full genome available (Table S1) to build a haplotype network (Fig. 5c). These data show that the various alleles at this locus are structured into two deeply divergent haplotypes, separated by at least 12 mutations, corresponding to the ones defined previously. Interestingly, the *GLR2.7* ^TCA^ haplotype displays high intra‐haplotype divergence as shown by the presence of rare alleles. This is potentially indicative of relaxed selection or drift occurring in some accessions. The broad geographical presence of both haplotypes and their deep divergence suggests that they might be ancient polymorphisms and have been conserved through selection. Several statistical tests – such as Tajima's *D* and Fu and Li's *F* or *D* – have been described to test the hypothesis that a sequence evolves neutrally by comparing the amount of variation observed and the variation expected for a given sequence. Deviations from neutrality are identified by either positive or negative test values, indicating balancing or purifying selection, respectively. Tajima's *D* is known to be sensitive to the species demography, and we therefore also computed the Fu and Li's *D* and *F* statistics (Tajima, 1989; Fu & Li, 1993), which incorporate an outgroup sequence (here *Arabidopsis lyrata*) to better discriminate signatures of selection from demographic history. All three tests displayed positive values on most parts of the *GLR2.7* gene sequence (Fig. 5d) with some stretches reaching significance, therefore clearly indicating that this locus does not evolve neutrally. Altogether, these results show that two deeply diverged haplotypes of *GLR2.7* segregate in natural Arabidopsis populations, and we provide evidence that balancing selection may be maintaining variation at this locus.

As *LecRK‐I.1*, the other locus identified in our GWAS experiment, also displays signatures of balancing selection with two dominant haplotypes (Groux *et al*., 2021), we looked at the distribution of strong and weak haplotypes from both genes between accessions. Interestingly, although they are not linked genetically (LD, *r* ^2^ = 0.025), an additive effect of haplotypes on HR‐like could be observed. Indeed, accessions harboring a strong haplotype for both genes (*GLR2.7* ^ATT^ and *LecRK‐I.1* ^TACAA^) showed a significantly higher symptom score than accessions harboring a weak haplotype (*GLR2.7* ^TCA^ and *LecRK‐I.1* ^CGTGC^), whereas accessions having a mixed combination displayed an intermediate phenotype (Fig. S5). On the contrary, EE‐induced SA was only correlated with the *GLR2.7* haplotype (Fig. S5), as expected by the lack of association at the *LecRK‐I.1* locus for this trait (Fig. S2).

### GLR2.7 expression

**Fig. 6:**
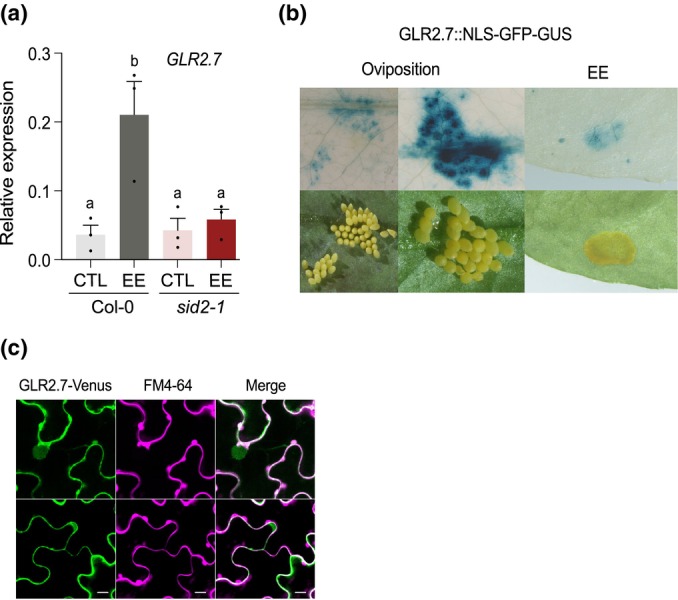
Expression of Arabidopsis *GLUTAMATE‐LIKE RECEPTOR2.7 (GLR2.7)* is induced by *Pieris brassicae* eggs. (a) *GLR2.7* expression in response to egg extract (EE) in Columbia (Col‐0) and salicylic acid deficient line *sid2‐1*. Transcript levels were monitored 3 d after treatment by real‐time quantitative polymerase chain reaction and normalized to the reference gene SAND. Mean ± SE of three technical replicates is shown. This experiment was repeated twice with similar results. Letters denote statistical differences (ANOVA followed by Tukey's HSD). (b) Beta‐glucuronidase (GUS) staining of GLR2.7::NLS‐GFP‐GUS line 3 d after *P. brassicae* oviposition or EE treatment. (c) Subcellular localization of transiently expressed UBQ10::GLR2.7‐Venus and the plasma membrane marker FM4‐64 in *Nicotiana benthamiana* leaves. Bars, 20 μm.

In order to explore how *GLR2.7* could contribute to defense responses, we first measured its expression after EE treatment. Expression of *GLR2.7* was significantly induced by EE, and this induction was dependent on SA, as shown by a lack of induction in *sid2‐1* (Fig. 6a). Additionally, analysis of previously published microarray data showed that *GLR2.7* expression is induced after natural oviposition by *P. brassicae* (Little *et al*., 2007). In line with these findings, we showed using a *GLR2.7*::NLS‐GFP‐GUS reporter line that *GLR2.7* expression is strongly induced at the site of *P. brassicae* oviposition or EE treatment (Fig. 6b).

To study the protein cellular localization, we expressed a Venus‐tagged *GLR2.7* under the strong UBQ10 promoter in *Nicotiana benthamiana* leaves. Most of the Venus signal was localized to the plasma membrane, as indicated by colocalization with the plasma membrane marker FM4‐64 (Fig. 6c). We, however, also noted signal at the nucleus and in cytosolic projections, which might be due to overexpression or indicate that GLR2.7 is not exclusively targeted to the plasma membrane.

Then, to assess whether *GLR2.7* expression was associated with symptom scores measured in the GWAS analysis, we selected 40 accessions from the mapping population, 21 with a weak (< 0.55) and 18 with a strong symptom score (> 2.55; Table S1), and we assessed *GLR2.7* expression in response to *P. brassicae* EE treatment. We observed variable basal and inducible levels of expression between accessions (Fig. S6; Table S1). Although basal expression was significantly higher in strong accessions, this difference was no longer significant after EE treatment (Fig. S6b). However, when looking at the *GLR2.7* haplotype in these 40 accessions, both basal and EE‐inducible *GLR2.7* expression were significantly higher in accessions harboring the *GLR2.7* ^ATT^ strong haplotype (Fig. S6b). Altogether, these findings point to a potential contribution of *GLR2.7* expression to the difference in egg‐induced responses between accessions.

Although *GLR2.7* is located next to *GLR2.8* and *GLR2.9*, we did not detect significant SNPs associated with HR‐like or SA levels in the two homologous genes. We anyhow observed a significant induction of the three genes in response to EE treatment, pointing to a potential additive role for GLR2.8 and GLR2.9 (Fig. S7a). However, single *glr2.8* and *glr2.9* knockout lines only showed a mild and nonsignificant reduction of EE‐induced cell death compared with the other lines, contrary to the reduction observed in *glr2.7* and *glr2.7/2.8/2*.9 lines compared with Col‐0 (Fig. S7b).

### GLR2.7 regulates egg‐induced cytosolic Ca^2+^ accumulation

**Fig. 7:**
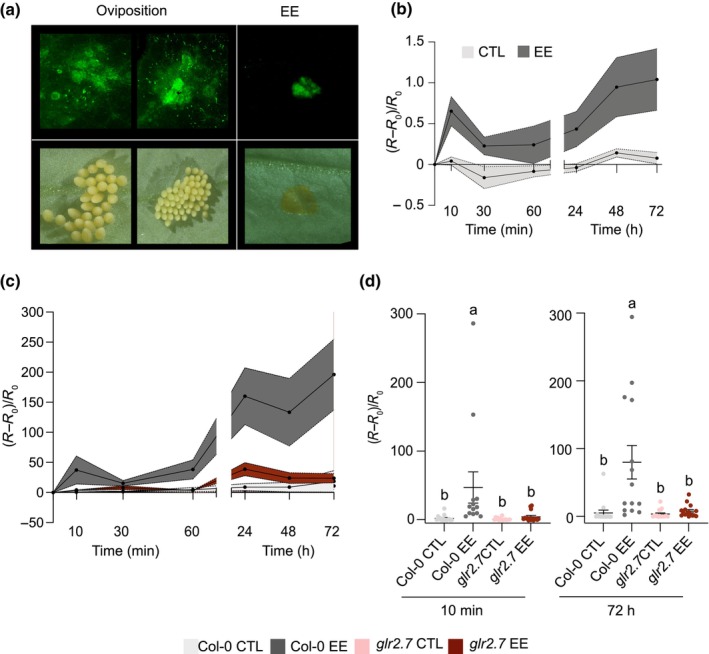
Clade 2 Arabidopsis glutamate receptor‐like proteins (GLRs) participate in egg‐induced cytosolic calcium (Ca^2+^) accumulation. (a) Visualization of cytosolic Ca^2+^ on the leaf adaxial side using Arabidopsis reporter GCaMP6f (ratiometric CGf line) 3 d after *Pieris brassicae* oviposition or egg extract (EE) treatment (upper panels). Corresponding pictures of the abaxial side are shown in the lower panels. (b) Time course of cytosolic calcium (Ca^2+^) accumulation after EE treatment in GCaMP6f plants. CTL, untreated. Mean ± SE of two biological replicates is shown (*n* = 6 per experiment). Values represents the ratio (*R*) of green fluorescence (GCaMP6f) to magenta fluorescence (mCherry; proportional to the Ca^2+^ concentration), normalized to the initial ratio (*R*_0_). (c) Time course of cytosolic Ca^2+^ accumulation after EE treatment in Columbia (Col‐0) or *glr2.7* containing GCaMP6f (MCGf and MCGf_*glr2.7* lines). Mean ± SE of one biological replicate is shown (*n* = 8). This experiment was repeated once with similar results. (d) Cytosolic Ca^2+^ quantification 10 min and 72 h after EE treatment. Values are mean ± SE from two independent biological replicates (*n* = 8 per experiment). Letters denote statistical differences (ANOVA followed by Tukey's HSD).

Members of the GLR gene family are amino acid‐gated Ca^2+^‐permeable channels that play a role in diverse physiological processes, including defense (Grenzi *et al*., 2022; Simon *et al*., 2023). Seminal studies have demonstrated the involvement of Arabidopsis clade 3 GLR 3.3 and GLR 3.6 in insect‐ and wound‐induced systemic electrical and Ca^2+^ signaling (Mousavi *et al*., 2013; Toyota *et al*., 2018; Gao *et al*., 2023). We thus tested whether eggs or EE application trigger cytosolic Ca^2+^ influx, potentially indicative of GLR function. We used a ratiometric Ca^2+^ reporter line CGf, which contains a mCherry reference domain fused to the intensiometric Ca^2+^ reporter GCaMP6f (Weigand *et al*., 2021). Strikingly, we observed a green fluorescent signal under *P. brassicae* eggs or at the site of EE application, indicating a localized cytosolic Ca^2+^ accumulation (Fig. 7a). A time‐course analysis showed that EE triggers a fast and long‐lasting cytosolic Ca^2+^ increase, with an early peak at 10 min followed by a gradual increase up to 72 h after application (Fig. 7b).

To specifically assess the contribution of GLR2.7, we introduced the CGf ratiometric reporter in Col‐0 and *glr2.7*. In the mutant line, Ca^2+^ influx was significantly reduced compared with Col‐0, pointing to a prominent role of GLR2.7 in EE‐induced Ca^2+^ accumulation (Fig. 7c,d).

We also took advantage of a genetically encoded YC3.6 Ca^2+^ indicator line with a large deletion in the GLR2.7‐GLR2.9 genomic region (Bjornson *et al*., 2021). Compared with YC3.6 Col‐0, the YC3.6 *glr2.7/2.8/2.9* triple‐mutant line accumulated significantly less Ca^2+^ after EE treatment, confirming the involvement of GLR2.7 in this response (Fig. S8).

**Fig. 8:**
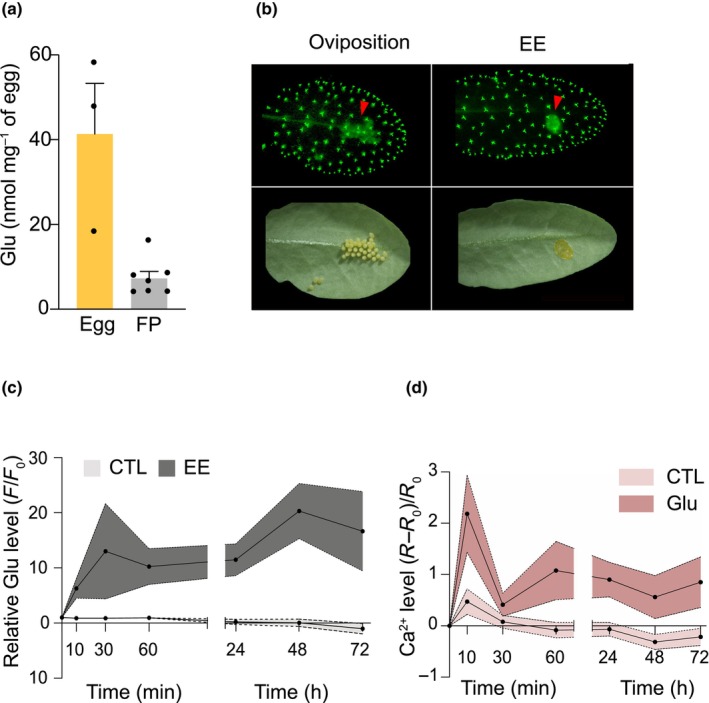
Egg‐derived glutamate (Glu) diffuses to Arabidopsis leaf surface and external Glu triggers glutamate receptor‐like protein (GLR)‐dependent calcium (Ca^2+^) influx. (a) Glu quantification in *Pieris brassicae* eggs and on filter paper (FP) below the eggs. Values are mean ± SE measured 1 d after oviposition (*n* = 3–7) (b) Visualization of apoplastic glutamate (red arrowheads) 3 d after oviposition or egg extract (EE) treatment using Arabidopsis reporter iGluSnFR (upper panels). Corresponding pictures of the leaf abaxial side are shown in the lower panels. Trichomes display constitutive Glu accumulation. (c) Time course of apoplastic Glu accumulation after EE treatment. Fluorescent signal (*F*) was measured in iGluSnFR and normalized to the value at time 0 (*F*_0_). Mean ± SE of two biological replicates is shown (*n* = 3 per experiment). (d) Time course of cytosolic Ca^2+^ accumulation after 10 mM Glu treatment in GCaMP6f plants. CTL, control solution (0.05% Silwet L‐77). Mean ± SE of two biological replicates is shown (*n* = 6 per experiment).

Glu is one of the known ligands for plant GLRs (Alfieri *et al*., 2019), and apoplastic Glu was shown to trigger systemic Ca^2+^ accumulation and defense responses (Toyota *et al*., 2018). We thus reasoned that insect eggs or egg‐associated secretions may contain and release Glu to activate plasma‐membrane localized GLRs. Strikingly, we measured 41.3 ± 11.9 nmol mg^−1^ of free Glu in eggs and 7.3 ± 1.6 nmol mg^−1^ on filter paper 1 d after *P. brassicae* oviposition, indicating that this amino acid either diffuses out of the egg or is present in egg‐associated secretions, and thus reaches the leaf surface during natural egg laying (Fig. 8a). We also found that, besides Glu, the majority of amino acids present in eggs are not detected on filter paper, with the exception of proline and, to a lesser extent, histidine and aspartic acid (Fig. S9). Then, to see whether exogenous Glu can enter the apoplastic space, we used the apoplastic Glu reporter line 35S::CHIB‐iGluSnFR (Toyota *et al*., 2018). Upon oviposition or EE treatment, we could clearly detect a fluorescent signal under the eggs or at the site of treatment, strongly suggesting the presence of apoplastic Glu (Fig. 8b). As a positive control, external application of 10 mM Glu, which is in the range found in EE, similarly induced a fluorescent signal at the site of treatment (Fig. S10a).

Also, quantification of the fluorescence signal over time indicated a fast and long‐lasting accumulation of apoplastic Glu in response to EE treatment (Fig. 8c). We then showed that Glu treatment triggers cytosolic Ca^2+^ increase at the site of application (Fig. S10b) and that the temporal accumulation (Fig. 8d) is similar to the one observed with EE treatment (Fig. 7). Finally, like with EE treatment (Fig. S8), the YC3.6 *glr2.7/2.8/2.9* triple mutant line accumulated significantly less Ca^2+^ after Glu treatment than YC3.6 Col‐0 (Fig. S10c,d).

Altogether, these findings thus identify GLR2.7 as a key component mediating egg‐induced defense responses through Ca^2+^ signaling and point to a potential activation via the presence of Glu at the oviposition site.

## Discussion

The use of natural variation helps reveal unprecedented levels of detail in the interactions between plants and insects by studying traits whose genetic variation has been shaped through selection and drift. The understanding of plant responses to insect eggs is still scarce at the molecular level but could provide useful tools for pest control (Tamiru *et al*., 2015; Fatouros *et al*., 2016). Here, we showed that mild cell death or chlorosis triggered by egg perception is associated with a new locus in Arabidopsis.

It is quite fascinating to observe such extensive variation in the response to *P. brassicae* eggs and EE in natural Arabidopsis accessions. Together with many phenotypes previously described, such as cell death, ROS, and SA accumulation, gene expression, and the induction of systemic acquired resistance (SAR; Little *et al*., 2007; Bruessow *et al*., 2010; Gouhier‐Darimont *et al*., 2013, 2019; Hilfiker *et al*., 2014), this observation further supports the findings that EE gives a similar, yet accelerated, response to real *P. brassicae* eggs (Little *et al*., 2007) and thus represents a useful tool to dissect this response. It is, however, noteworthy that Arabidopsis accessions develop a range of relatively mild symptoms compared with other Brassicaceae, like *B. nigra*, that display a strong egg‐killing necrosis (Bonnet *et al*., 2017; Griese *et al*., 2020, 2021). Although Arabidopsis and *B. nigra* trigger cell death, defense gene expression and SA accumulation (Shapiro & DeVay, 1987; Bonnet *et al*., 2017; Griese *et al*., 2020; Caarls *et al*., 2023), these illustrate a generic PTI response that is activated by EAMP perception. The enhanced ETI‐like response of some Brassicaceae species likely illustrates the ongoing arms race between adapted herbivores and resistant plants that have evolved genes to counteract effectors, including the *PEK* locus that contains TNL receptor genes (Bassetti *et al*., 2024). However, given recent findings suggesting that PTI and ETI immune pathways are interconnected (Yuan *et al*., 2021a,b), explaining similarities in downstream responses, future studies should aim at clarifying whether Arabidopsis lacks resistance genes to oviposition.

In this study, we provide clear evidence that the *GLR2.7* locus identified by the GWAS mapping modulates egg‐induced PTI. Indeed, the transfer of *GLR2.7* from strongly responsive accessions to weakly responsive ones can restore chlorosis, higher SA accumulation and higher ROS production. This finding is corroborated by decreased EE‐induced symptoms and defenses in a *glr2.7* knockout line in Col‐0. The large effect associated with the *GLR2.7* locus in the accession panel most likely indicates that it is an upstream component of the EE‐induced response, consistent with the strong association observed with total SA levels. Despite being mildly correlated, both traits thus appear to have a partially overlapping genetic structure. Since variation in symptoms has an additional contribution from *LecRK‐I.1* (Groux *et al*., 2021), these findings reveal that the response of Arabidopsis to EE of *P. brassicae* has a polygenic structure, with two loci accounting for a large proportion of the variation observed. This is consistent with the observation that quantitative disease resistance is usually under the control of multiple genes (Roux *et al*., 2014; Corwin & Kliebenstein, 2017) and contrasts with the *B. nigra* HR‐like response that is under the control of the single locus *PEK* (Bassetti *et al*., 2024).

Although it is located next to *GLR2.8* and *GLR2.9*, *GLR2.7* alone can rescue the phenotype of a weak accession, and EE‐induced Ca^2+^ influx is significantly reduced in *glr2.7*. In addition, EE‐induced cell death was not affected in *glr2.8* and *glr2.9* mutants. It is thus unlikely that these two homologs have a substantial contribution to egg‐induced defenses, although it cannot be formally ruled out. Further investigation will be needed to clarify this point. Recently, *GLR2.7*, *GLR2.8* and *GLR2.9* were shown to belong to a set of core immunity responsive genes, which were induced by various pathogen‐associated molecular patterns (PAMPs) involved in PTI and, consistently, the *glr2.7/2.8/2*.9 mutant was more susceptible to bacterial infection (Bjornson *et al*., 2021). Thus, studying the various roles of each member of this subset of clade 2 GLRs may unveil specific or overlapping functions in response to different biotic stresses.

Interestingly, we observed that the *GLR2.7* locus consists mainly of two diverged haplotypes present throughout the entire Arabidopsis population studied. Both haplotypes are found at intermediate frequency independently of any geographic or phylogenetic pattern, strongly suggesting that balancing selection contributes to their long‐term maintenance (Wu *et al*., 2017). This hypothesis is further supported by the significant presence of positive Tajima's *D* and Fu and Li's *D*/*F* statistics in the coding sequence of GLR2.7. Remarkably, similar signatures of selection were previously observed at immune loci in Arabidopsis (Todesco *et al*., 2010; Huard‐Chauveau *et al*., 2013; Karasov *et al*., 2014) and in the *Capsella* genus (Koenig *et al*., 2019). Given that GLR2.7 was identified in a process related to direct defenses against insect eggs, it is conceivable that the presence of a strong haplotype could confer a selective advantage in natural Arabidopsis populations exposed to insect oviposition by promoting stronger HR‐like. The recent finding that GLR2.7 also functions in response to pathogens (Bjornson *et al*., 2021), together with the absence of any clear geographical separation of the haplotypes, suggests that microenvironment variations in herbivore and/or pathogen pressure may structure this gene. As both *GLR2.7* and *LecRK‐I.1* show signatures of balancing selection but haplotypes are not linked genetically, further research should aim at understanding the reasons for the maintenance of such genetic polymorphisms in natural populations.

### The potential role of GLR2.7 in egg‐induced responses

The family of *GLUTAMATE RECEPTOR‐LIKE* genes was first described based on their homology to animal ionotropic glutamate receptors (iGluRs), yet phylogenetic analyses indicate that plant and animal GLRs diverged from a common ancestor. As opposed to iGluRs in animals, which are mainly involved in neurotransmission, plant GLRs play roles in various developmental processes and stress responses (Simon *et al*., 2023). In particular, GLR3 clade members have been previously reported to be involved in pathogen‐ and herbivore‐triggered immunity (Li *et al*., 2013; Manzoor *et al*., 2013; Mousavi *et al*., 2013; Xue *et al*., 2022), whereas GLR2 members are much less characterized. Plant GLRs are generally regarded as amino acid‐gated Ca^2+^‐permeable channels (Wudick *et al*., 2018), although in most cases, ligands are not known. In comparison with animal iGluRs, data gathered so far on plant GLRs indicate that they have a broader specificity and that, besides Glu, the ligand binding site can accommodate other amino acids (Alfieri *et al*., 2019).

Previous transcriptome studies revealed that genes related to Ca^2+^ transport and signaling were upregulated in response to oviposition (Little *et al*., 2007; Lortzing *et al*., 2019; Ojeda‐Martinez *et al*., 2021) but, to our knowledge, there is not yet evidence for Ca^2+^ accumulation. We found that *P. brassicae* oviposition and EE treatment trigger a localized and long‐lasting cytosolic Ca^2+^ accumulation and that this response was substantially reduced in the *glr2.7* mutant. This strongly suggests a link between Ca^2+^ signaling and egg‐induced defense responses and supports the involvement of GLR2.7. Given that the Ca^2+^ influx was not fully abolished in *glr2.7* or *glr2.7/2.8/2.9* lines, other GLRs or other types of Ca^2+^ channels may contribute to the response and will deserve further investigation. The localization of GLR2.7 to the plasma membrane and a strong upregulation of *GLR2.7* expression underneath the eggs provide additional evidence for a role of this channel in the modulation of egg‐induced responses.

Strikingly, we also showed that Glu that originates from *P. brassicae* eggs or egg‐associated secretions accumulates in the apoplastic space following oviposition or EE treatment. Also, exogenously applied Glu entered rapidly in the apoplastic space and triggered Ca^2+^ accumulation. One plausible explanation is that exogenous Glu could act as a GLR2.7 ligand to activate the channel, although we cannot formally discard the possibility that eggs induce the release of cytosolic Glu in the apoplastic space. This process has been shown to occur following PAMP or pathogen perception and leaf injury (Vatsa *et al*., 2011; O'Leary *et al*., 2016; Grenzi *et al*., 2023). *In vitro* and *in vivo* studies showed that L‐Cys, L‐Glu and Gly have strong affinity for GLR3.3 and induce Ca^2+^ responses in roots (Alfieri *et al*., 2019). Another study on GLR2.9 revealed that Gly is a probable ligand of the channel and that Gly‐mediated Ca^2+^ influx was inhibited by the animal iGluRs antagonist DNQX, suggesting a potentially conserved function in amino acid sensing (Dubos *et al*., 2003). However, we did not detect Cys nor Gly on filter paper after egg deposition, suggesting that they are unlikely to activate GLR2.7 in the context of oviposition. But, again, whether eggs induce the extracellular accumulation of different amino acids that bind GLR2.7 will clearly deserve further investigation. Also, testing the effect of other potential amino acid ligands on calcium channel activity of GLR2.7 would be interesting.

Intriguingly, out of 49 SNPs significantly associated with symptom score, four are located in the *GLR2.7* promoter and 16 lead to amino acid changes to the protein sequence. The significant correlation between haplotype and *GLR2.7* expression suggests that SNPs in the promoter may affect the binding of transcription factors or that SNPs in the gene sequence may modulate expression via modification of mRNA secondary structure, miRNA target sites or mRNA stability. This hypothesis will require a systematic analysis of the impact of single or multiple SNPs on *GLR2.7* expression. Alternatively, changes in amino acids may alter GLR2.7 function. A structure of Arabidopsis GLR3.4 has identified a ligand‐binding domain for Glu as well as the transmembrane helices important for ion channel assembly (Green *et al*., 2021). Interestingly, the predicted amino acid substitutions K669L, K670T, E520G, A533T, and V544T are in close proximity to the Glu‐binding domain, whereas K672Q and K673R are within the ion channel. Moreover, the substitution D448V (SNP3) is located in the linker between the activation and ligand‐binding domains. Whether these residues are crucial for ligand‐binding and channel activity will need further studies of biochemically reconstituted GLR2.7 variants in heterologous systems (Alfieri *et al*., 2019; Green *et al*., 2021). These findings anyhow suggest that variation in egg‐induced responses may be regulated at different levels.

The discovery of Glu as a potential modulator of egg‐induced defenses, through activation of GLR2.7, adds to our recent finding that PCs in lepidopteran eggs trigger immune responses that partially require LecRK‐I.8 (Stahl *et al*., 2020). Although no genetic variation was found at the *LecRK‐I.8* locus for egg‐induced HR‐like, such variation was identified for the close homolog LecRK‐I.1 (Groux *et al*., 2021). This suggests a complex regulatory process whereby the potential perception of egg‐derived PC by both cell‐surface receptors leads to a downstream signaling cascade whose strength may depend on *LecRK‐I.1* haplotypes. In addition, the modulation of this response by GLR2.7 provides another layer of fine‐tuning, supported by the additive role of *GLR2.7* and *LecRK‐I.1* haplotypes. The exact mechanism by which GLR2.7‐dependent Ca^2+^ influx modulates PC/LecRKI.1/8‐dependent signaling is currently unknown. The association of GLR2.7 with egg‐induced SA accumulation and the fact that SA regulates downstream HR‐like responses and defense gene expression (Gouhier‐Darimont *et al*., 2013) suggest a role in the early steps following egg perception. Decoding of Ca^2+^ signals is mediated by Ca^2+^ sensors, including calmodulins, calcineurin B‐like proteins, and calcium‐dependent protein kinases. Research in PTI signaling has revealed a complex and flexible regulation of cellular responses to Ca^2+^, connecting decoders with various transcription factors, including CALMODULIN BINDING PROTEIN 60 g and SYSTEMIC ACQUIRED RESISTANCE DEFICIENT 1 that control SA biosynthesis (Zheng *et al*., 2015; Xu *et al*., 2022; Jiang & Ding, 2023).

In conclusion, we have identified GLR2.7 as an important component of the insect egg perception pathway in Arabidopsis, highlighting a novel role for a member of clade 2 GLRs. Future studies should focus on the activation mechanism of GLR2.7 and Ca^2+^ influx in response to oviposition. To place our results in a biological context, future studies should aim at testing the quantitative contribution of GLR2.7 to egg hatching/survival and whether it also impacts further larval performance. It will also be important to monitor oviposition‐induced calcium accumulation in accessions with different GLR2.7 haplotypes and explore the ecological significance of conserved haplotypes at the locus.

## Competing interests

None declared.

## Author contributions

MM, RG and PR conceived and designed the research. RG, MM, CG‐D, PM and CAMR conducted experiments. MM, RG and PR analyzed the data. PR, RG and MM wrote the manuscript with input from all authors. MM and RG contributed equally to this work.

## Disclaimer

The New Phytologist Foundation remains neutral with regard to jurisdictional claims in maps and in any institutional affiliations.

## Supporting information


**Fig. S1** CRISPR–Cas9 targeting of Arabidopsis *GLR2.7*.
**Fig. S2** Manhattan plot of GWAS mapping for SA accumulation.
**Fig. S3** Complementation of Ull2‐3 weak accession with *GLR2.7*.
**Fig. S4** Geographic distribution of *GLR2.7* polymorphisms across Europe.
**Fig. S5** Contribution of *GLR2.7* and *LecRKI.1* haplotypes.
**Fig. S6** *GLR2.7* expression in accessions with weak or strong symptom scores.
**Fig. S7** Role of GLR2.7 homologs.
**Fig. S8** Calcium influx in *glr2.7/2.8/2.9*.
**Fig. S9** Amino acid release from *Pieris brassicae* eggs or egg‐associated secretions.
**Fig. S10** Glutamate accumulates in the apoplastic space and triggers Ca^2+^ influx.

**Table S1** SNPs in the *GLR2.7* locus that are significantly associated with symptom score.
**Table S2** List of primers used in this study.
**Table S3** Significantly associated substitutions in the Arabidopsis GLR2.7 protein sequence.
Please note: Wiley is not responsible for the content or functionality of any Supporting Information supplied by the authors. Any queries (other than missing material) should be directed to the *New Phytologist* Central Office.

## Data Availability

All data that support our findings are available as Table S1.
