# Supplementary material for: The glutamate receptor‐like GLR2.7 modulates insect egg‐induced defense responses in Arabidopsis
Source: New Phytol. 2025 Jul 26;248(2):897–912. doi: 10.1111/nph.70405 (PMC12445815; doi:10.1111/nph.70405)
Supplement: Supplementary file 1 — Fig. S1 CRISPR–Cas9 targeting of Arabidopsis GLR2.7. Fig. S2 Manhattan plot of GWAS mapping for SA accumulation. Fig. S3 Complementation of Ull2‐3 weak accession with GLR2.7. Fig. S4 Geographic distribution of GLR2.7 polymorphisms across Europe. Fig. S5 Contribution of GLR2.7 and LecRKI.1 haplotypes. Fig. S6 GLR2.7 expression in accessions with weak or strong symptom scores. Fig. S7 Role of GLR2.7 homologs. Fig. S8 Calcium influx in glr2.7/2.8/2.9. Fig. S9 Amino acid release from Pieris brassicae eggs or egg‐associated secretions. Fig. S10 Glutamate accumulates in the apoplastic space and triggers Ca2+ influx. [file NPH-248-897-s002.docx]

## *New Phytologist* Supporting Information

Article title: The glutamate receptor-like GLR2.7 modulates insect egg-induced defense responses in Arabidopsis

Authors: Maria Mineiro, Raphaël Groux, Caroline Gouhier-Darimont, Pierre Mateo, Christelle Aurélie Maud Robert and Philippe Reymond

Article acceptance date: 03 July 2025

The following Supporting Information is available for this article:

**Fig. S1** CRISPR–Cas9 targeting of Arabidopsis *GLR2.7*

**Fig. S2** Manhattan plot of GWAS mapping for SA accumulation

**Fig. S3** Complementation of Ull2-3 weak accession with *GLR2.7*

**Fig. S4** Geographic distribution of *GLR2.7* polymorphisms across Europe

**Fig. S5** Contribution of *GLR2.7* and *LecRK-I.1* haplotypes

**Fig. S6** *GLR2.7* expression in accessions with weak or strong symptom scores

**Fig. S7** Role of GLR2.7 homologues

**Fig. S8** Calcium influx in *glr2.7/2.8/2.9*

**Fig. S9** Amino acid release from *Pieris brassicae* eggs or egg-associated secretions

**Fig. S10** Glutamate accumulates in the apoplastic space and triggers Ca^2+^ influx

**Table S1** SNPs in the *GLR2.7* locus that are signficantly associated with symptom score

**Table S2** List of primers used in this study

**Table S3** List of all significantly associated substitutions in the GLR2.7 protein sequence


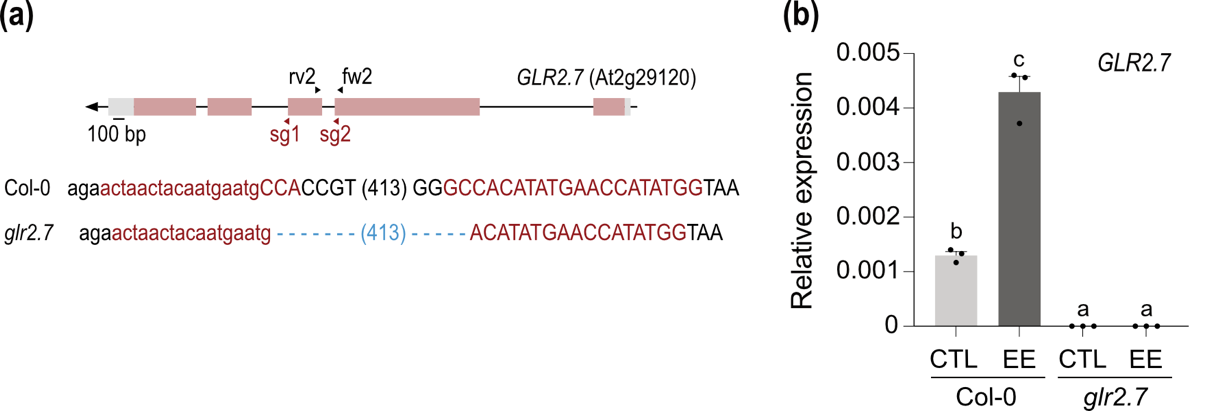


**Fig. S1 CRISPR–Cas9 targeting of Arabidopsis *GLR2.7*.** (a) Shown is the *GLR2.7* locus. Pink boxes, black lines and gray boxes represent exonic, intronic and untranslated regions, respectively. Single guides are indicated with red arrowheads. Single-guide sequences are indicated in red letters; deletions are indicated with blue dashes; sequence gap lengths are given in parentheses. RT-QPCR primers are indicated by black arrowheads. (b) Expression of *GLR2.7* after 3 days of *Pieris brassicae* EE treatment. Transcript levels were monitored by RT-qPCR and normalized to the reference gene *SAND*. Mean ± SE of three technical replicates is shown. Letters denote statistical differences (ANOVA followed by Tukey's HSD).

**
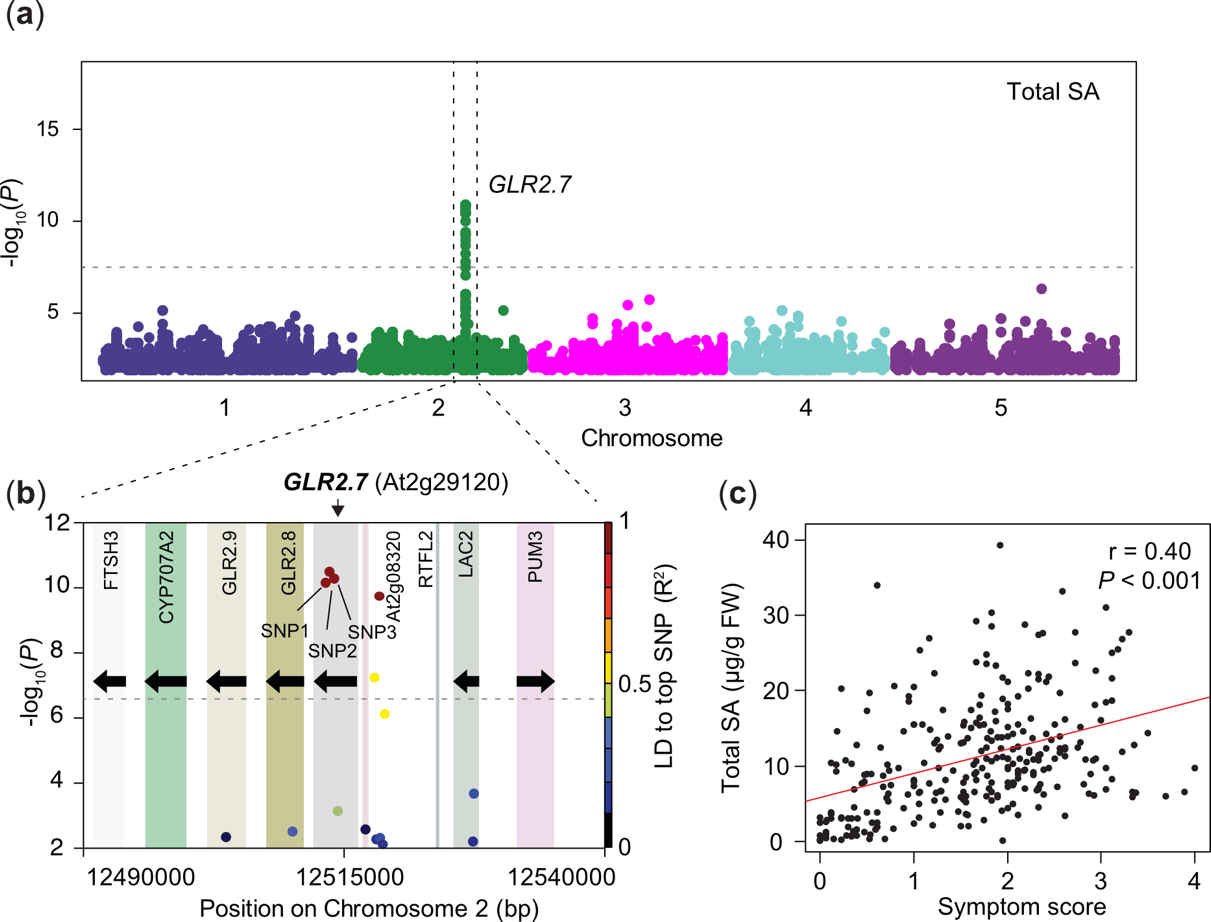
**

**Fig. S2 Manhattan plot of GWAS mapping for SA accumulation** (a) Full imputed genotypes for all 295 *Arabidopsis thaliana* accessions was used for mapping using an accelerated mixed-model. Chromosomes are displayed in different colors and the dashed line indicates the Bonferroni-corrected significance at threshold α=0.05. (b) Local association plot of the GLR2.7 locus using the 250K genotype data for symptom score. The x-axis represents genomic position on chromosome 2 and color boxes indicate genes. LD with the most significant SNP is indicated by a color scale. The dashed line indicates the Bonferroni corrected significance threshold at α=0.05. (c) Population-wide relationship between total SA levels and symptoms score after 5 days of *Pieris brassicae* EE treatment. The red line indicates linear regression trend line, Pearson correlation coefficient and correlation significance are shown.

**
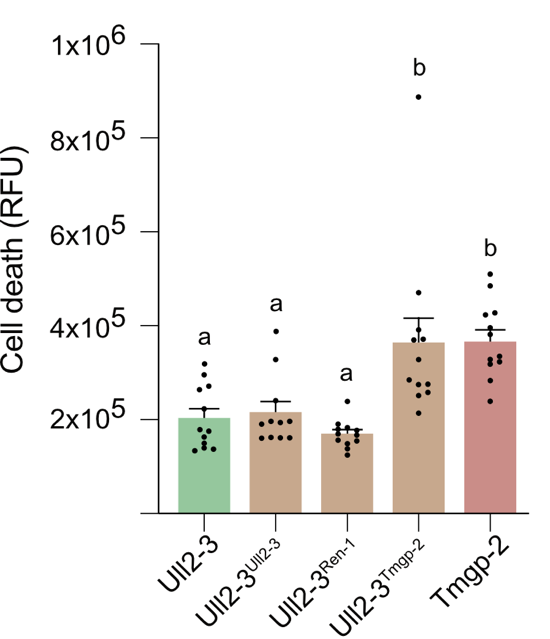
**

**Fig. S3 Complementation of Ull2-3 weak accession with *GLR2.7*.** *Arabidopsis thaliana* ecotype Ull2-3 was transformed with *GLR2.7* from Ull2-3 (ULL2-3^Ull2-3^) and Ren-1 (Ull2-3^Ren-1^) weak accessions, and with GLR2.7 from Tomegap2 (Ull2-3^Tmgp-2^) strong accession. Cell death quantification after 6 days of *Pieris brassicae* egg extract (EE) treatment was measured by red light fluorescence. Mean ± SE of one biological replicate is shown (n=10-12) , this experiment was repeated once with similar results. Letters denote statistical differences (ANOVA followed by Tukey's HSD).

**
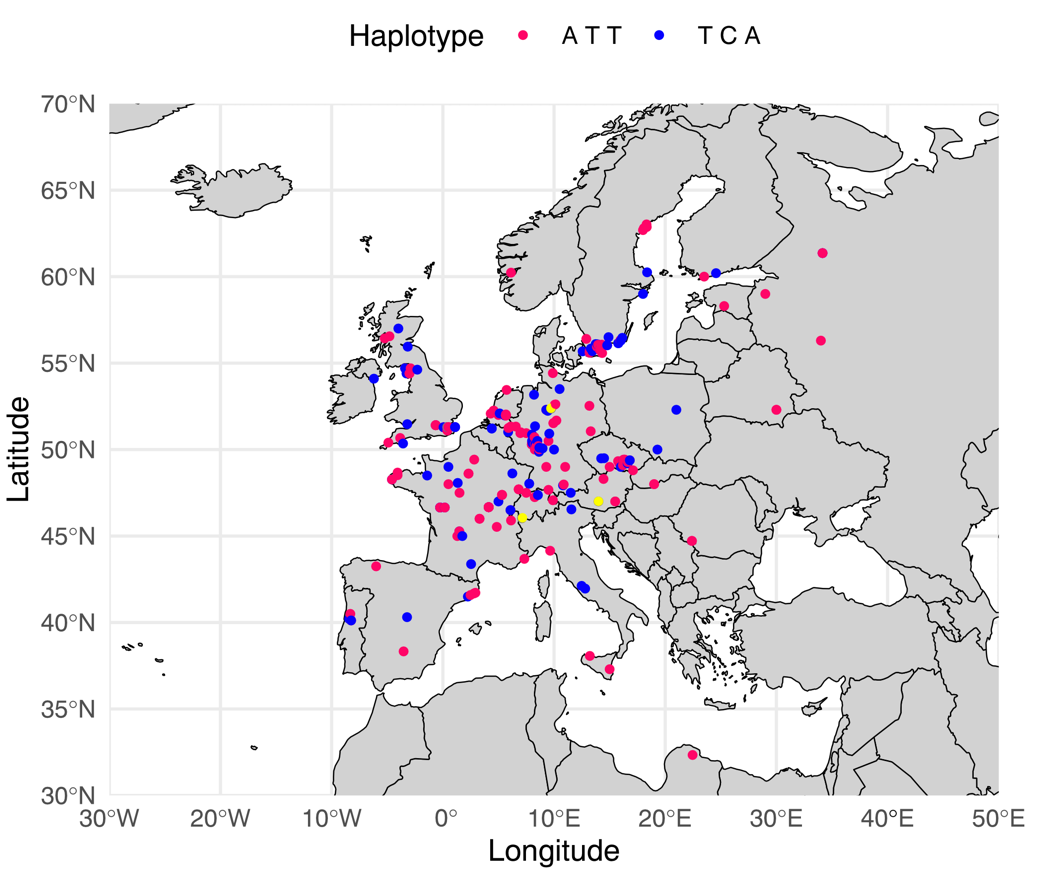
**

**Fig. S4 Geographic distribution of *GLR2.7* polymorphisms across Europe.** Shown here is the spatial distribution of the two major *GLR2.7* haplotypes, GLR2.7^ATT^ (magenta dots) that is associated with strong HR-like in response to *Pieris brassicae* EE, and GLR2.7^TCA^ (blue dots) that is associated with weak HR-like. Each dot represents a distinct *Arabidopsis thaliana* ecotype used in the GWAS study. Yellow dots, other haplotypes.

**
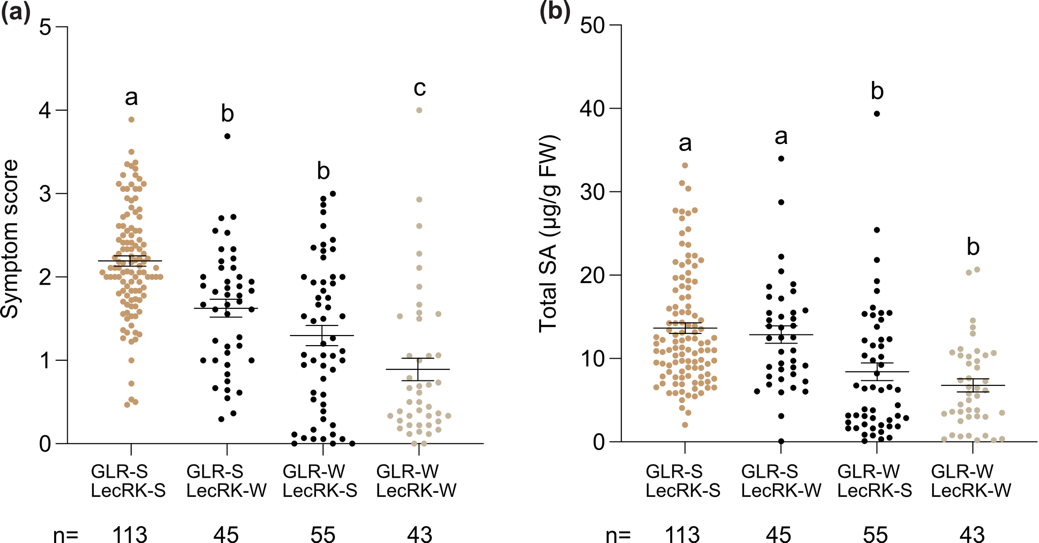
**

**Fig. S5 Contribution of *GLR2.7* and *LecRK-I.1* haplotypes.** Symptom score (a) and total SA (b) (mean ± SE) of *Arabidopsis thaliana* accessions based on the presence of *GLR2.7* and *LecRK-I.1* haplotypes that are associated with strong (S) or weak (W) HR-like. Plants were treated for 5 days with *Pieris brassicae* EE. Each accession is represented by a dot. Letters denote statistical differences (ANOVA followed by Tukey’s HSD). GLR-S (GLR2.7^ATT^), GLR-W (GLR2.7^TCA^), LecRK-S (LecRK-I.1^TACAA^), LecRK-W (LecRK-I.1^CGTGC^).

**
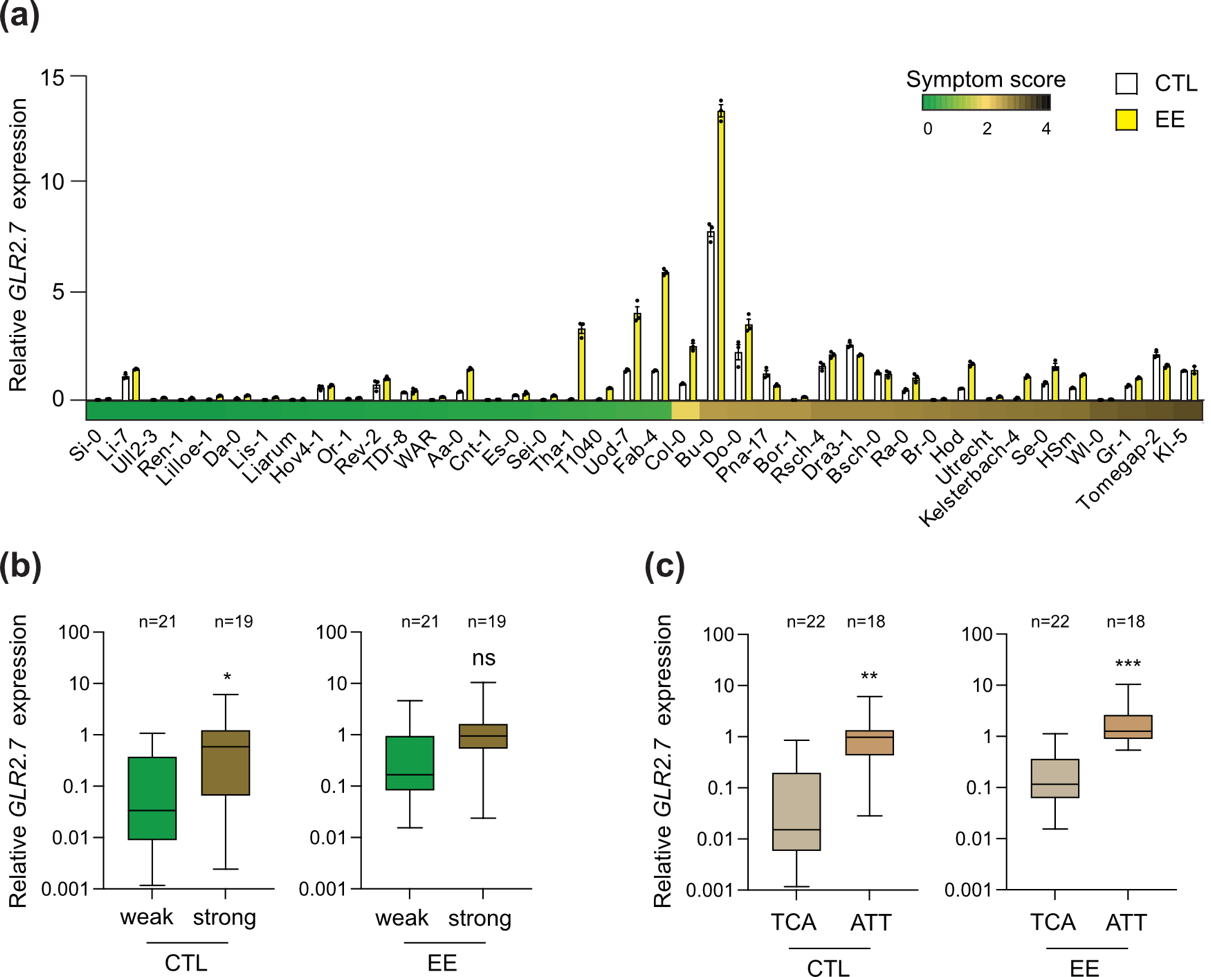
**

**Fig. S6 *GLR2.7* expression in accessions with weak or strong symptom scores.** (a) Transcript levels were monitored 3 days after *Pieris brassicae* EE treatment by RT-qPCR and normalized to the reference gene SAND. Mean ± SE of three technical replicates is shown. (b,c) Box and whisker plots of *GLR2.7* expression between *Arabidopsis thaliana* accessions with weak or strong symptom scores (b) or between accessions with different *GLR2.7* haplotypes (c). Asterisks denote statistical differences (Student's *t* test, **P*<0.05, ***P*<0.01, ****P*<0.001, ns, not significant.

**
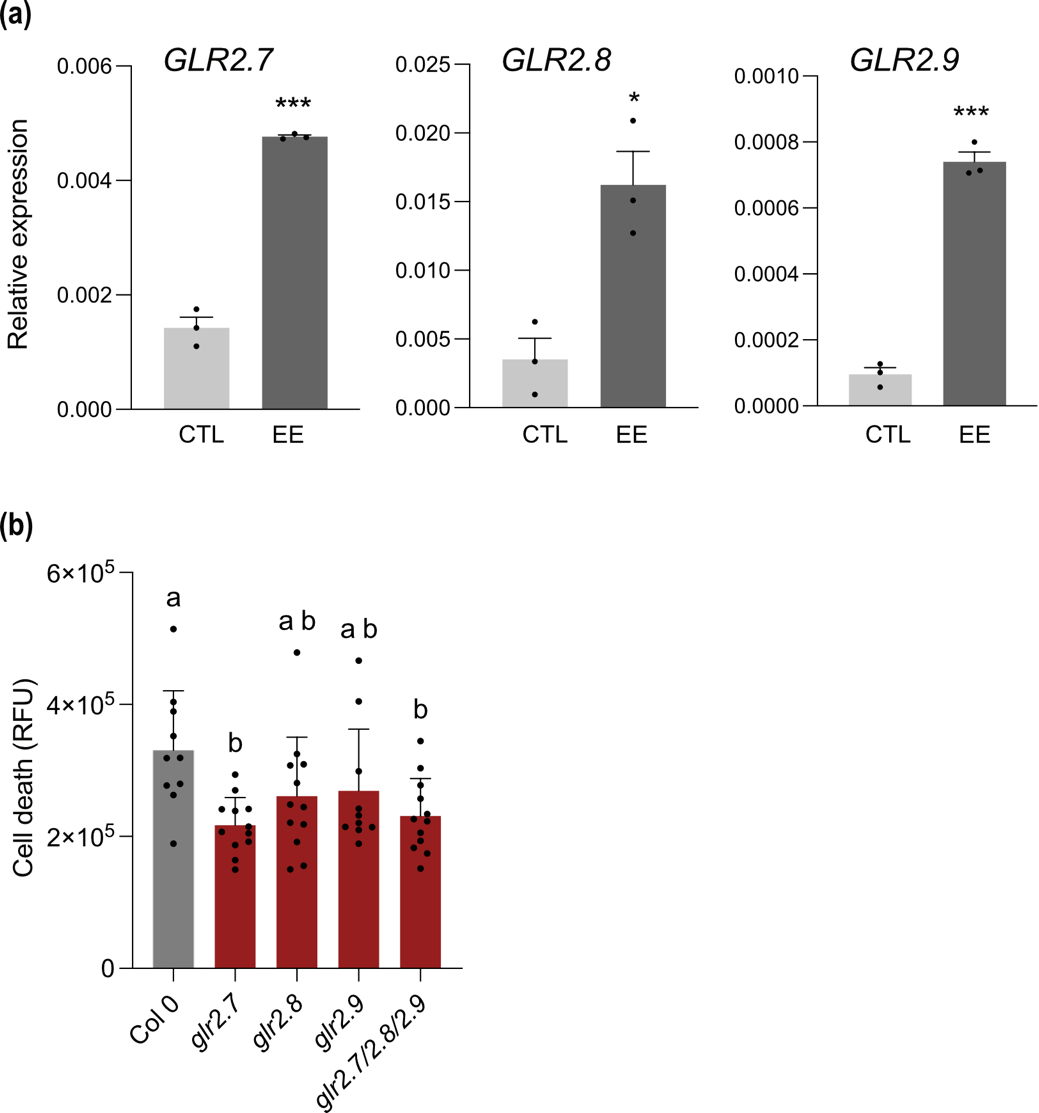
**

**Fig. S7 Role of GLR2.7 homologues.** (a) Expression of Arabidopsis *GLR2.7*, *GLR2.8* and *GLR2.9* in response to *Pieris brassicae* egg extract (EE). Transcript levels were monitored 3 days after treatment by RT-qPCR and normalized to the reference gene SAND. Mean ± SE of three technical replicates is shown. This experiment was repeated twice with similar results. Asterisks denote statistical differences (Student's *t*-test, **P*<0.05, ****P*<0.001). (b) Cell death quantification after 6 days of EE treatment was measured by red light fluorescence. Mean ± SE of one biological replicate is shown (n=12). This experiment was repeated once with similar results. Letters denote statistical differences (ANOVA followed by Tukey’s HSD). Single mutants are T-DNA insertion lines. The triple mutant was generated by CRISPR-Cas9.

**
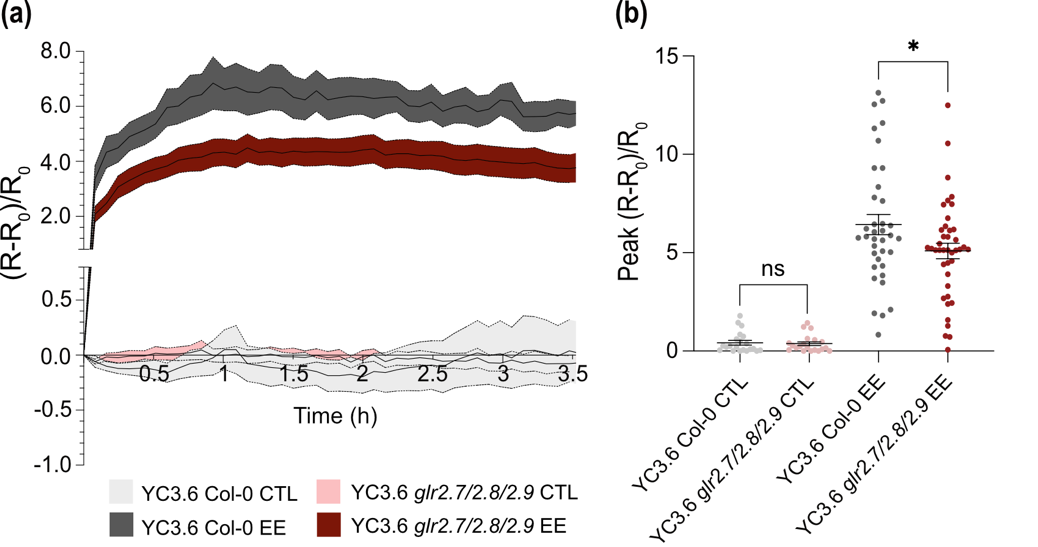
**

**Fig. S8 Calcium influx in *glr2.7/2.8/2.9*** (a) Cytosolic Ca^2+^ quantification after *Pieris brassicae* EE treatment in Arabidopsis YC3.6 seedling (Col-0 or *glr2.7/2.8/2.9* background). Mean ± SE of one biological replicate is shown, this experiment was repeated twice with similar results (n=10-16 per experiment). Values represents the ratio (R) of yellow fluorescence (YFP) to cyan fluorescence (CFP) (proportional to the Ca^2+^ concentration), normalized to the initial ratio (R_0_). (b) Peak value of the Ca^2+^ influx described in panel (a). Values are mean ± SE from three independent biological replicates. Statistical differences between genotypes are indicated (Student's *t*-test, **P*< 0.05).

**
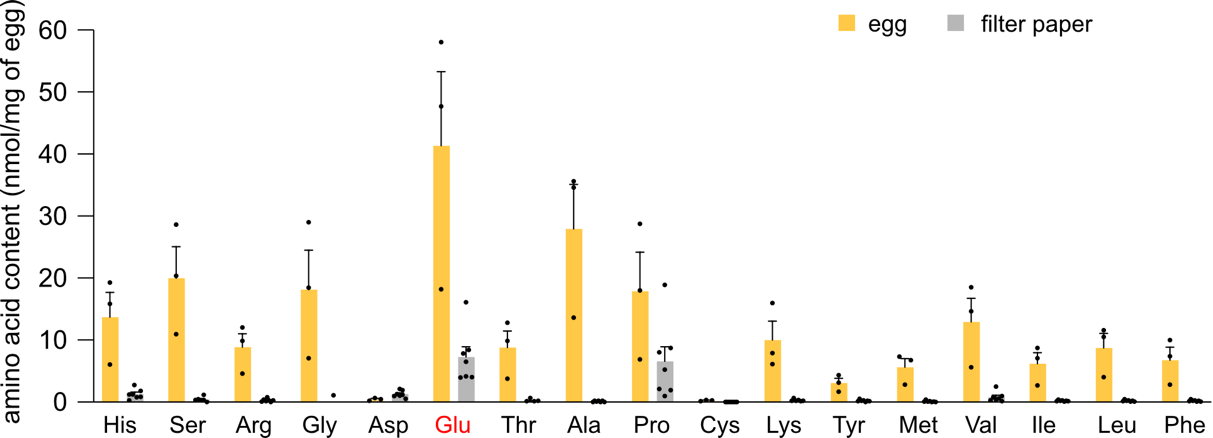
**

**Fig. S9 Amino acid release from *Pieris brassicae* eggs or egg-associated secretions.** Butterflies oviposited on filter paper and, after one day, amino acid levels were quantified in eggs and on filter paper. Mean ± SE of one biological replicate is shown (n=3-7). This experiment was repeated once with similar results.

**
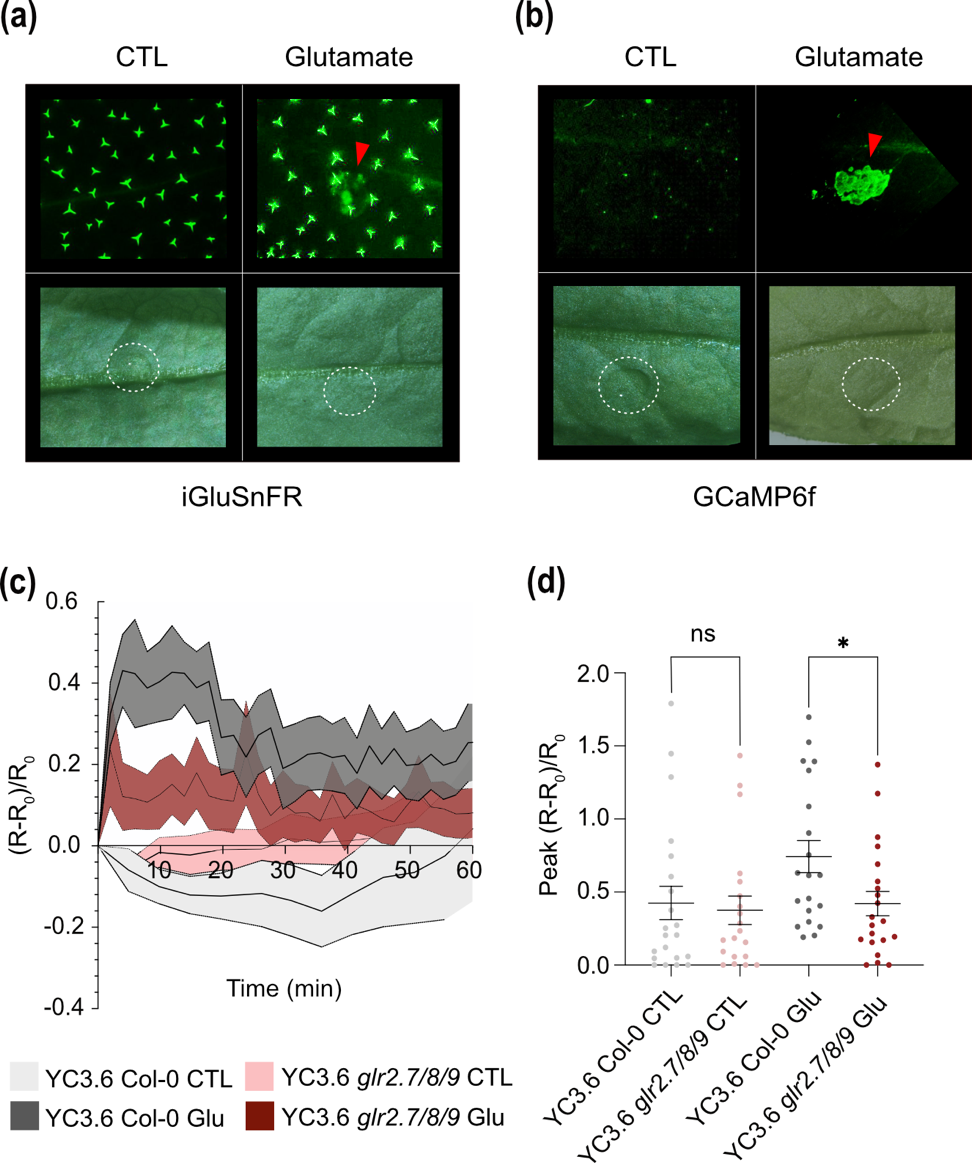
**

**Fig. S10 Glutamate accumulates in the apoplastic space and triggers Ca^2+^ influx.** (a) Visualization of apoplastic glutamate (red arrowhead, top panel) 1 hr after 10 mM glutamate treatment using Arabidopsis reporter iGluSnFR. Trichomes display constitutive Glu accumulation. CTL, control solution (0.05% Silwet L-77). (b) Visualization of cytosolic Ca^2+^ (red arrowhead, top panel) 1 hr after 10 mM glutamate treatment using Arabidopsis reporter GCaMP6f. Dotted circle, site of application. Corresponding pictures of the leaf abaxial side are shown in the lower panels. (c) Cytosolic Ca^2+^ quantification after 10 mM Glu treatment in Arabidopsis YC3.6 seedling (Col-0 or *glr2.7/2.8/2.9* background). Mean ± SE of one biological replicate is shown, this experiment was repeated twice with similar results (n=10-16 per experiment). Values represents the ratio (R) of yellow fluorescence (YFP) to cyan fluorescence (CFP) (proportional to the Ca^2+^ concentration), normalized to the initial ratio (R_0_). (d) Peak value of the Ca^2+^ influx described in panel (c). Values are mean ± SE from three independent biological replicates. Statistical differences between genotypes are indicated (Student's *t*-test, **P*< 0.05)

**Table S2** List of primers used in this study.

Gene ID Primer Sequence

**Genotyping**

GLR2.7 At2g29120 G GLR2.7 fw GGAAATCTTGCCGGTTAAAAG

G GLR2.7 rv ACAAATTTGGGGACATTAGGG

CCglr2.7 fw2 GTAACGCAATGACCCCAACG

CCglr2.7 rv2 ATGGGACTCGTTTCCTAGCG

**Cloning**

GLR2.7 promoter GLR2.7p fw TTATAAGAGACTTCCGCAATGT

GLR2.7p rv CCAGAATTGAGGAACTTTATGTC

CRISPR-Cas9 cc_GLR_7a_fw (sg2) GGTATACCAAGTATACACCG

cc_GLR_7b_rev (sg1) ACCGTAAGTAACATCAATCA

GCaMP6f attB1_Fragment_FOR ACAAGTTTGTACAAAAAAGCAGGCT

GCaMP6-rv AGCTGGAGCTGAATTCCCG

GLR2.7 Tomegap-2 and Lz-0

GLR2.7-clon-Fw GATGCAACTTTTTTAAAGGGGG

GLR2.7-clon-Rv ATTGCCTAACAATTCCGTTATG

GLR2.7-EcoRV-Fw CCGCGGATATCGATGCAACTTTTTTAAAGGGGG

GLR2.7-BamHI-Rv CGGGATCCCGGATTGCCTAACAATTCCGTTATG

Ren-1 and Ull2-3

GLR2.7-clon-Fw2 CGATGTCTCAAGGGAAATCTTGAAT

GLR2.7-5UTR-Rv CCAGATTGAGGAACTTTATGTCAT

GLR2.7-ATG-Fw ATGAAAGTGATGAACCCTAGAAAAAC

GLR2.7-clon-Rv ATTGCCTAACAATTCCGTTATG

Venus GLR2.7-5UTR-Fw CTTAGTAGGATATTCACTAGCGTTAAAA

GLR2.7-clon-Rv ATTGCCTAACAATTCCGTTATG

**RT-qPCR**

SAND At2g28390 SAND fw AACTCTATGCAGCATTTGATCCACT

SAND rv TGATTGCATATCTTTATCGCCATC

PR1 At2g14610 PR1 fw GTGGGTTAGCGAGAAGGCTA

PR1 rv ACTTTGGCACATCCGAGTCT

SAG13 At2g29350 SAG13 fw GTCGTGCATGTCAATGTTGG

SAG13 rv CCAAGGACAAACAGAGTTCG

GLR2.7 At2g29120 Q GLR2.7 fw2 CC TCTTGTCTCCAGATGAGAAC

Q GLR2.7 rv2 CC GTACGGTAGCGTGAAATC

Q_GLR2.7_acc_fw GTGGCCTACATTAAAGTTATCC (for accessions)

Q_GLR2.7_acc_rev CAATATGTTGCATTTCTTCGCC (for accessions)

GLR2.8 At2g29110 GLR2.8_fw CAATACTGCTCCAAATATGCC

GLR2.8_rev CCTGTCAAAGGTGAATTCCTG

GLR2.9 At2g29100 GLR2.9_fw TCAATCCTGCTCTAAATATGTC

GLR2.9_rev CTCGATTTGTTGCGTTACATTG

**Table S3.** Significantly associated substitutions in the Arabidopsis GLR2.7 protein sequence. Non-synonymous substitutions as compared to Col-0 reference allele are indicated in bold. Alt, alternative allele.

Position on Chr.2 Col-0/Alt Substitution

12511933 cCa/cAa **P802Q**

12512197 tCc/tGc **S749C**

12512383 tTt/tAt **F687Y**

12512425 cTg/cGg **L673R**

12512429 Aaa/Caa **K672Q**

12513017 agC/agT S582

12513027 aGa/aAa **R579K**

12513065 (**SNP2**) gtA/gtG V566

12513122 agG/agA R547

12513133 Gta/Ata **V544I**

12513134 atA/atC I543

12513164/12513166 GcC/AcG **A533T**

12513316/12513317 gAG/gGC **E520G**

12513390 Ata/Gta **I496V**

12513391 gaG/gaC **E495D**

12513467 aAa/aCa **K470T**

12513470/12513471 AAg/TTg **K469L**

12513472 gtG/gtA V468

12513495 Atg/Ttg **M461L**

12513499 ggA/ggG G459

12513533 (**SNP3**) gAt/gTt **D448V**

12513601 aaG/aaA K425

12514453 acA/acT T141

12515737 Aag/Cag **K53Q**
